# Recurrent De Novo Dominant Mutations in *SLC25A4* Cause Severe Early-Onset Mitochondrial Disease and Loss of Mitochondrial DNA Copy Number

**DOI:** 10.1016/j.ajhg.2016.11.001

**Published:** 2016-12-01

**Authors:** Kyle Thompson, Homa Majd, Cristina Dallabona, Karit Reinson, Martin S. King, Charlotte L. Alston, Langping He, Tiziana Lodi, Simon A. Jones, Aviva Fattal-Valevski, Nitay D. Fraenkel, Ann Saada, Alon Haham, Pirjo Isohanni, Roshni Vara, Inês A. Barbosa, Michael A. Simpson, Charu Deshpande, Sanna Puusepp, Penelope E. Bonnen, Richard J. Rodenburg, Anu Suomalainen, Katrin Õunap, Orly Elpeleg, Ileana Ferrero, Robert McFarland, Edmund R.S. Kunji, Robert W. Taylor

(The American Journal of Human Genetics *99*, 860–876; October 6, 2016)

In the originally published version of this article, Cristina Dallabona’s first name was unfortunately misspelled. It appears correctly here and online. The authors regret the error.

