# Supplementary material for: Recurrent De Novo Dominant Mutations in SLC25A4 Cause Severe Early-Onset Mitochondrial Disease and Loss of Mitochondrial DNA Copy Number
Source: Am J Hum Genet. 2016 Sep 29;99(4):860–76. doi: 10.1016/j.ajhg.2016.08.014 (PMC5065686; doi:10.1016/j.ajhg.2016.08.014)
Supplement: Document S1. Figures S1–S3 and Table S1 [file mmc1.pdf]

The American Journal of Human Genetics, Volume *99*

Supplemental Data

Recurrent De Novo Dominant Mutations in *SLC25A4* Cause Severe Early-Onset Mitochondrial Disease and Loss of Mitochondrial DNA Copy Number

Kyle Thompson, Homa Majd, Christina Dallabona, Karit Reinson, Martin S. King, Charlotte L. Alston, Langping He, Tiziana Lodi, Simon A. Jones, Aviva Fattal-Valevski, Nitay D. Fraenkel, Ann Saada, Alon Haham, Pirjo Isohanni, Roshni Vara, Inês A. Barbosa, Michael A. Simpson, Charu Deshpande, Sanna Puusepp, Penelope E. Bonnen, Richard J. Rodenburg, Anu Suomalainen, Katrin Õunap, Orly Elpeleg, Ileana Ferrero, Robert McFarland, Edmund R.S. Kunji, and Robert W. Taylor

Supplemental Information

Supplemental Figures

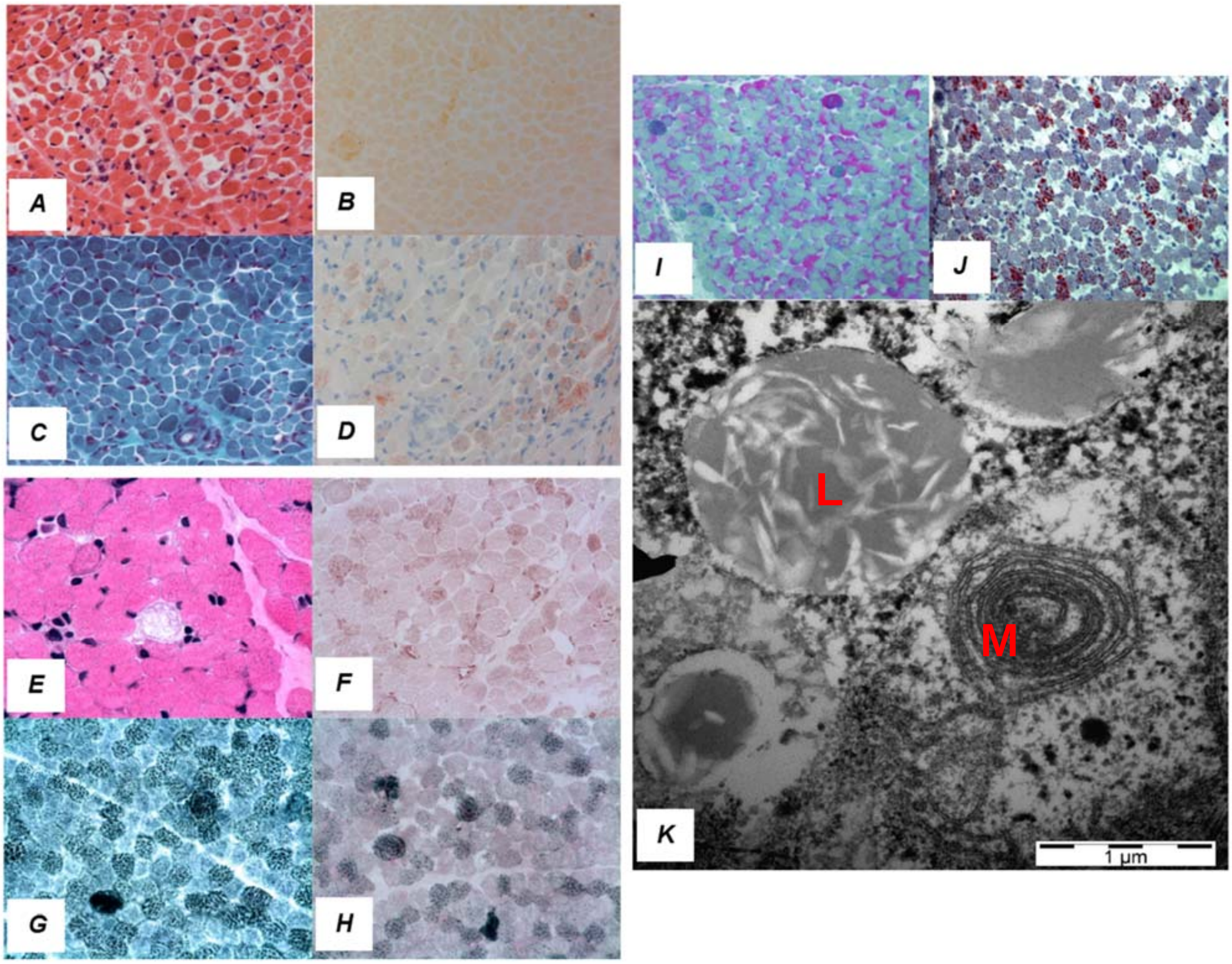

Figure S1: Muscle histopathology and electron microscopy in subjects with de novo heterozygous c.239G>A, p.(Arg80His) *SLC25A4* mutation
Histopathological analysis of a serially-sectioned skeletal muscle biopsy from Subject 1 showing (***A***) H&E staining, (***B***) cytochrome *c* oxidase (COX) reaction, (***C***) modified Gomori Trichrome staining and (***D***) Oil Red O staining highlighting evidence of weak COX reactivity across the section and accumulation of neutral lipid. Similar findings are evident in subject 2 based on (***E***) H&E staining, (***F***) cytochrome *c* oxidase (COX) reaction, (***G***) succinate dehydrogenase reaction showing fibres with enhanced mitochondrial numbers and (***H***) Sudan black staining to identify lipid deposition. Whilst oxidative enzyme reactions were not undertaken in subject 3, (***I***) modified Gomori trichrome staining and (***J***) Oil Red O staining show abnormal accumulations respectively. (***K***) An electron micrograph shows abnormal mitochondria with circularly arranged cristae (**M**) and lipid droplets (**L**).

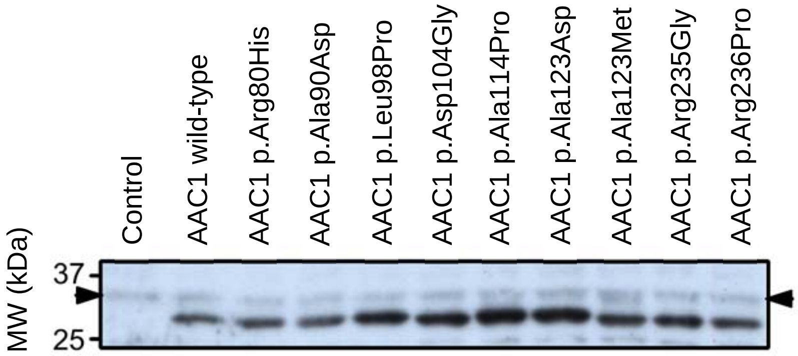

Figure S2: Recombinant AAC1 mutant proteins expressed in *L. lactis*
Western blot showing expression of the various human AAC1 mutants associated with disease. The arrow head shows a non-specific band, which serves as a loading control.

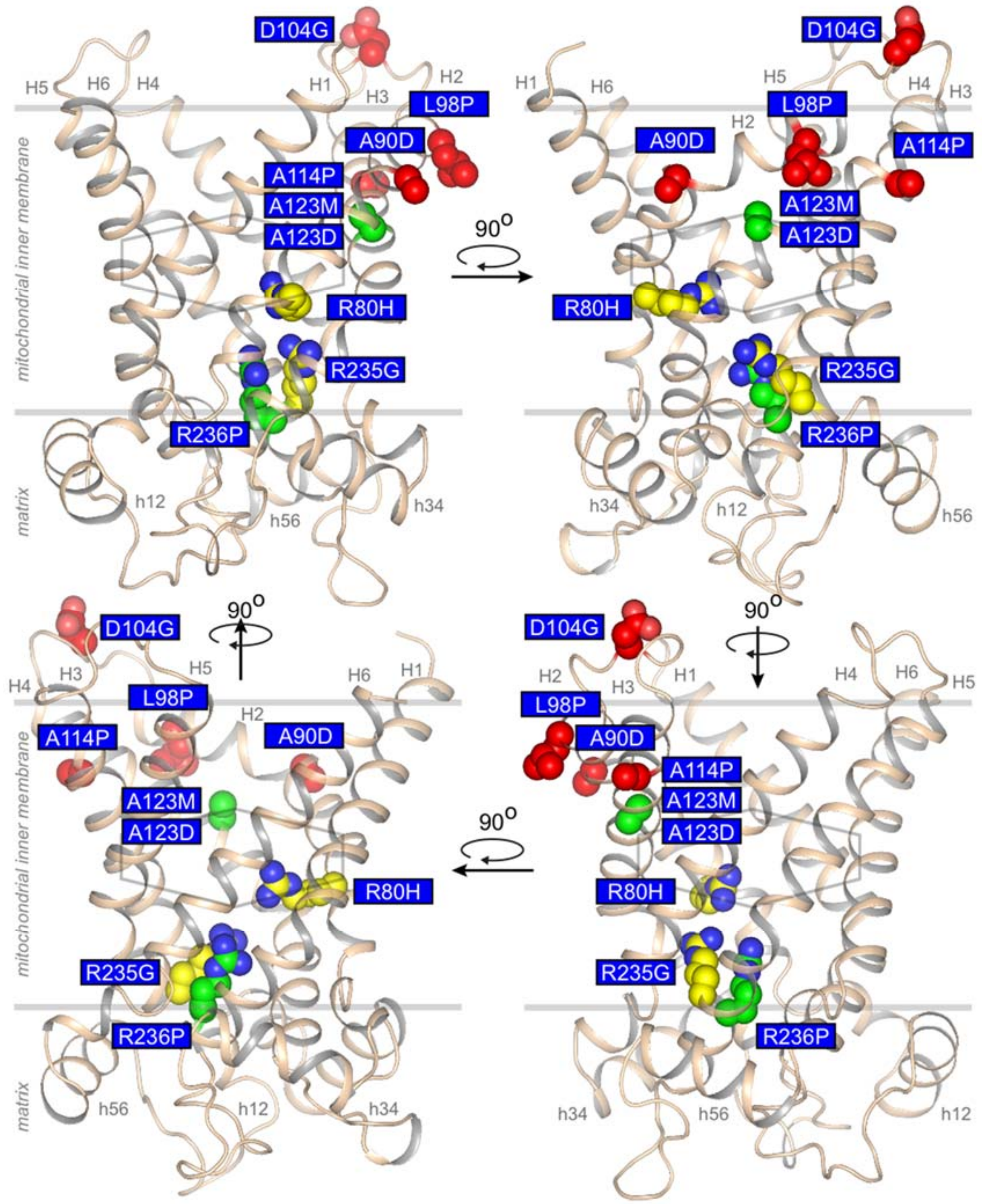

Figure S3: Position of disease-associated AAC1 mutations

Lateral view of the human ADP/ATP carrier from the membrane, showing the location of residues found to be mutated in disease. The *de novo* mutations are shown in yellow, dominant adPEO mutations in red and the recessive mutations in green.

Supplemental Table

Table S1: Previously reported *SLC25A4* mutations and associated clinical phenotype			
**Inheritance**	**Mutation**	**Clinical Phenotype**	**Reference**
Dominant	c.[342 G>C] + [=]<br>p.[(Ala114Pro)] + [=]	adPEO	Kaukonen *et al.* 2000
Dominant	c.[867 G>A] + [=]<br>p.[(Val289Met)] + [=]	adPEO	Kaukonen *et al.* 2000
Dominant	c.[293T>C] + [=]<br>p.[(Leu98Pro)] + [=]	adPEO	Napoli *et al.* 2001
Dominant	c.[311A>G] + [=]<br>p.[(Asp104Gly)] + [=]	adPEO	Komaki *et al.* 2002
Dominant	c.[293T>C] + [=]<br>p.[(Leu98Pro)] + [=]	adPEO,	Siciliano *et al.* 2003
Dominant	c.[269C>A] + [=]<br>p.[(Ala90Asp)] + [=]	adPEO	Deschauer *et al.* 2005
Recessive	c.[368C>A] + c.[368C>A]<br>p.[(Ala123Asp)] + p.[(Ala123Asp)]	Mitochondrial myopathy and cardiomyopathy	Palmieri *et al.* 2005
Recessive	c.[111+1G>A] + c.[111+1G>A]<br>(abolishes GT splice donor site of intron 1)	Mitochondrial myopathy and cardiomyopathy	Echaniz-Laguna *et al.* 2012
Recessive	c.[523delC] + c.[523delC]<br>p.[(Gln175ArgfsX38)] + p.[(Gln175ArgfsX38)]	Mitochondrial myopathy and cardiomyopathy	Strauss *et al.* 2013
Recessive	c.[707G>C] + c.[116-137del]<br>p.[(Arg236Pro)] + p.[(Gln39Leufs*14)]	Mitochondrial myopathy and cardiomyopathy	Korver-Keularts *et al.* 2015

Supplemental References:

Kaukonen, J. et al. Role of adenine nucleotide translocator 1 in mtDNA maintenance. *Science* 2000:**289**, 782–785.

Napoli, L. et al. A novel missense adenine nucleotide translocator-1 gene mutation in a Greek adPEO family. *Neurology* 2001:**57**, 2295–2298.

Komaki, H. et al. A novel D104G mutation in the adenine nucleotide translocator 1 gene in autosomal dominant progressive external ophthalmoplegia patients with mitochondrial DNA with multiple deletions. *Ann Neurol*. 2002:**51**, 645–648.

Siciliano, G. et al. Autosomal dominant external ophthalmoplegia and bipolar affective disorder associated with a mutation in the ANT1 gene. *Neuromuscul. Disord*. 2003:**13**, 162–165.

Deschauer, M. et al. A novel ANT1 gene mutation with probable germline mosaicism in autosomal dominant progressive external ophthalmoplegia. *Neuromuscul. Disord.* 2005:**15**, 311–315.

Palmieri, L. et al. Complete loss-of-function of the heart/muscle-specific adenine nucleotide translocator is associated with mitochondrial myopathy and cardiomyopathy. *Hum Mol Genet* 2005:**14**, 3079–3088.

Echaniz-Laguna, A. et al. Complete loss of expression of the ANT1 gene causing cardiomyopathy and myopathy. *Journal of Medical Genetics* 2012:**49**, 146–150.

Strauss, K.A. et al. Severity of cardiomyopathy associated with adenine nucleotide translocator-1 deficiency correlates with mtDNA haplogroup. *PNAS* 2013:**110**, 3453–3458.

Körver-Keularts, I.M.L.W. et al. Two Novel Mutations in the SLC25A4 Gene in a Patient with Mitochondrial Myopathy. *JIMD Rep* 2015:**22**:39-
