# Supplementary material for: Recurrent De Novo Dominant Mutations in SLC25A4 Cause Severe Early-Onset Mitochondrial Disease and Loss of Mitochondrial DNA Copy Number
Source: Am J Hum Genet. 2016 Sep 29;99(4):860–76. doi: 10.1016/j.ajhg.2016.08.014 (PMC5065686; doi:10.1016/j.ajhg.2016.08.014)
Supplement: Document S2. Article plus Supplemental Data [file mmc2.pdf]

ARTICLE

Recurrent De Novo Dominant Mutations in *SLC25A4* Cause Severe Early-Onset Mitochondrial Disease and Loss of Mitochondrial DNA Copy Number

Kyle Thompson,[1,20] Homa Majd,[2,20] Christina Dallabona,[3,20] Karit Reinson,[4,5,20] Martin S. King,[2] Charlotte L. Alston,[1] Langping He,[1] Tiziana Lodi,[3] Simon A. Jones,[6] Aviva Fattal-Valevski,[7] Nitay D. Fraenkel,[8] Ann Saada,[9] Alon Haham,[10] Pirjo Isohanni,[11,12] Roshni Vara,[13] Inês A. Barbosa,[14] Michael A. Simpson,[14] Charu Deshpande,[15] Sanna Puusepp,[4,5] Penelope E. Bonnen,[16] Richard J. Rodenburg,[17] Anu Suomalainen,[11,18] Katrin Õunap,[4,5] Orly Elpeleg,[19] Ileana Ferrero,[3] Robert McFarland,[1] Edmund R.S. Kunji,[2,21] and Robert W. Taylor[1,21,*]

Mutations in *SLC25A4* encoding the mitochondrial ADP/ATP carrier AAC1 are well-recognized causes of mitochondrial disease. Several heterozygous *SLC25A4* mutations cause adult-onset autosomal-dominant progressive external ophthalmoplegia associated with multiple mitochondrial DNA deletions, whereas recessive *SLC25A4* mutations cause childhood-onset mitochondrial myopathy and cardiomyopathy. Here, we describe the identification by whole-exome sequencing of seven probands harboring dominant, de novo *SLC25A4* mutations. All affected individuals presented at birth, were ventilator dependent and, where tested, revealed severe combined mitochondrial respiratory chain deficiencies associated with a marked loss of mitochondrial DNA copy number in skeletal muscle. Strikingly, an identical c.239G>A (p.Arg80His) mutation was present in four of the seven subjects, and the other three case subjects harbored the same c.703C>G (p.Arg235Gly) mutation. Analysis of skeletal muscle revealed a marked decrease of AAC1 protein levels and loss of respiratory chain complexes containing mitochondrial DNA-encoded subunits. We show that both recombinant AAC1 mutant proteins are severely impaired in ADP/ATP transport, affecting most likely the substrate binding and mechanics of the carrier, respectively. This highly reduced capacity for transport probably affects mitochondrial DNA maintenance and in turn respiration, causing a severe energy crisis. The confirmation of the pathogenicity of these de novo *SLC25A4* mutations highlights a third distinct clinical phenotype associated with mutation of this gene and demonstrates that early-onset mitochondrial disease can be caused by recurrent de novo mutations, which has significant implications for the application and analysis of whole-exome sequencing data in mitochondrial disease.

Introduction

Mitochondria are essential organelles involved in a wide range of cellular processes, including iron-sulfur cluster formation, amino acid and fatty acid synthesis and degradation, the tricarboxylic acid cycle, heme synthesis, and production of ATP via oxidative phosphorylation (OXPHOS). Mitochondria contain their own genome (mtDNA) that encodes 2 rRNAs, 22 tRNAs, and 13 polypeptides,[1] all of which are hydrophobic subunits of the complexes involved in OXPHOS. The maintenance and expression of mtDNA and all other mitochondrial processes require many proteins that are encoded by the nuclear genome. Mitochondrial disease results from a disruption of this complex bi-genomic control of OXPHOS and may arise from a wide range of genetic defects, which, in turn, result in a vast array of clinical features. In these circumstances, achieving a genetic diagnosis can be challenging. However, the advent of next-generation sequencing has greatly improved the prospect of achieving a genetic diagnosis for affected individuals and the spectrum of mitochondrial disease-causing mutations continues to expand.[2] Whole-exome sequencing (WES) has identified many of these novel mutations[3] and sequencing parental samples

[1]Wellcome Trust Centre for Mitochondrial Research, Institute of Neuroscience, Newcastle University, Newcastle upon Tyne NE2 4HH, UK; [2]The Medical Research Council, Mitochondrial Biology Unit, Cambridge Biomedical Campus, Wellcome Trust/MRC Building, Hills Road, Cambridge CB2 0XY, UK; [3]Department of Life Sciences, University of Parma, Parco Area delle Scienze 11A, Parma 43124, Italy; [4]Department of Pediatrics, Institute of Clinical Medicine, University of Tartu, 51014 Tartu, Estonia; [5]Department of Genetics, United Laboratories, Tartu University Hospital, 51014 Tartu, Estonia; [6]Manchester Centre for Genomic Medicine, Central Manchester University Hospitals NHS Foundation Trust, St Marys Hospital, Oxford Road, Manchester M13 9WL, UK; [7]Paediatric Neurology Unit, "Dana" Children Hospital, Tel Aviv Sourasky Medical Centre, Sackler Faculty of Medicine, Tel Aviv University, 64239 Tel Aviv, Israel; [8]Department of Respiratory Rehabilitation, Alyn Hospital, Jerusalem 91090, Israel; [9]Metabolic Laboratory Department of Genetics and Metabolic Diseases, Hadassah-Hebrew University Medical Center, Jerusalem 91120, Israel; [10]Neonatal Intensive Care Unit, "Lis" Maternity Hospital, Tel Aviv Sourasky Medical Centre, 64239 Tel Aviv, Israel; [11]Research Programs Unit, Molecular Neurology, Biomedicum-Helsinki, University of Helsinki, 00290 Helsinki, Finland; [12]Department of Pediatric Neurology, Children's Hospital, Helsinki University Hospital and University of Helsinki, 00290 Helsinki, Finland; [13]Department of Paediatric Inherited Metabolic Diseases, Evelina Children's Hospital, London SE1 7EH, UK; [14]Division of Genetics and Molecular Medicine, King's College London School of Medicine, London SE1 9RY, UK; [15]Clinical Genetics Unit, Guys and St Thomas' NHS Foundation Trust, London SE1 9RT, UK; [16]Department of Molecular and Human Genetics, Baylor College of Medicine, Houston, TX 77030, USA; [17]Radboud Center for Mitochondrial Medicine, Department of Paediatrics, Translational Metabolic Laboratory, Radboud University Medical Centre, 6525 GA Nijmegen, the Netherlands; [18]Department of Neurosciences, Helsinki University Hospital and University of Helsinki, 00290 Helsinki, Finland; [19]The Monique and Jacques Roboh Department of Genetic Research, Hadassah-Hebrew University Medical Center, Jerusalem 91120, Israel
[20]These authors contributed equally to this work
[21]These authors contributed equally to this work
*Correspondence: robert.taylor@ncl.ac.uk
http://dx.doi.org/10.1016/j.ajhg.2016.08.014.
© 2016 The Author(s). This is an open access article under the CC BY license (http://creativecommons.org/licenses/by/4.0/).

in addition to the proband is an efficient means of determining segregation of suspected pathogenic variants.

Mitochondrial carriers represent a large group of nuclear-encoded mitochondrial proteins with diverse substrates. The transport steps performed by these carriers are required for the processes in mitochondria described above and for the replication, transcription, and translation of mtDNA.[4] They typically consist of three homologous sequence repeats[5] and cycle between two states: the cytoplasmic state in which the substrate-binding site is open to the intermembrane space and the matrix state in which the substrate-binding site is open to the mitochondrial matrix. An increasing number of mutations in genes encoding mitochondrial carriers have been reported to cause mitochondrial disease.[6]

One of these, the mitochondrial ADP/ATP carrier (AAC) imports ADP into the mitochondrion and exports ATP into the intermembrane space, which is confluent with the cytosol.[7] Given its pivotal role, it is not surprising that mutations in the ADP/ATP carrier are associated with mitochondrial disease.[8–16] The ADP/ATP carrier has four tissue-specific isoforms in humans, which are encoded by four closely related nuclear genes.[17] The isoform expressed at high levels in skeletal muscle, heart, and brain is AAC1 (also known as ANT1) and is encoded by *SLC25A4* (MIM: 103220) located on the sub-telomeric region of chromosome 4q.[18,19]

Several mutations in *SLC25A4* have been linked to mitochondrial disorders and fall into two distinct clinical phenotypes: null recessive mutations causing a mitochondrial myopathy and cardiomyopathy phenotype that presents in childhood or early adulthood and is characterized by fatigue and exercise intolerance (also described as mitochondrial DNA depletion syndrome type 12 [MIM: 615418])[13–16] and several single heterozygous mutations reported in cases of adult-onset autosomal-dominant progressive external ophthalmoplegia (adPEO [MIM: 609283]).[8–12] Here we present the clinical, biochemical, and functional characterization of previously undocumented, recurrent, de novo dominant mutations in *SLC25A4* identified by WES in seven affected individuals with a characteristic and severe early-onset mitochondrial disease presentation representing a third distinct phenotypic group associated with *SLC25A4* mutations.

Material and Methods

Muscle Histology and Biochemistry

Informed consent with appropriate ethics review committee approvals were obtained for all families. Histological and histochemical analyses were performed on 10 μm transversely orientated serial cryosections of skeletal muscle biopsy samples using standard procedures where available. The activities of individual respiratory chain complex activities and citrate synthase, a mitochondrial matrix marker, were determined in muscle homogenates for subjects 1, 2, and 5 as previously described.[20] Complex I, complex II+III, and complex IV activities were measured in subject 3 as previously described.[21] The respiratory chain enzyme activities CI + III, CII + III, CII, CIV, and citrate synthase were analyzed in subject 6 as previously described.[22]

Whole-Exome Sequencing

All subjects underwent routine mtDNA diagnostic testing including assessment of mtDNA copy number by qPCR and sequencing of the mitochondrial genome. Whole-exome capture, sequencing, and variant filtering/prioritization was performed for subject 1, as previously described.[3] WES was performed in the family trio of subject 2 using Agilent SureSelectXT All Exon v.5 according to manufacturer's instructions followed by sequencing on an Illumina HiSeq2500 platform with 100 bp paired-end reads. Variant calls were generated with an in-house pipeline as previously described[23] with minor alterations. In brief, resulting reads were aligned to the reference genome (hg19) with the Novoalign (Novocraft Technologies Sdn Bhd) alignment tool. Clonal reads resulting from polymerase chain reaction, optical errors, and reads mapping to multiple locations were discarded from further analyses. SNPs and small insertion deletions were identified and filtered for quality with SAMtools. Variant files were annotated with respect to genes and variant functional consequences using ANNOVAR tool. Further annotation included information on variant novelty and estimated population frequencies by cross-referencing identified variants with publically available data (dbSNP135, 1000 Genomes database) and >1,000 control exomes processed with the pipeline described above.

WES of subject 3 was performed as described previously.[24,25] Variants in genes with (predicted) mitochondrial function were filtered based on frequency in publically available databases (dbSNP, ExAC) and an in-house database, revealing the likely pathogenic heterozygous variant in *SLC25A4*. Identification of mitochondrial disease-causing variants was achieved by filtering data using publically available databases of genes with known or predicted mitochondrial function and of genes previously associated with related clinical phenotypes. Rare (minor allele frequency < 0.01) and previously unobserved alleles under both dominant and recessive models in each affected individual were evaluated.

For subjects 4 and 5, WES was performed as previously described.[26] For subject 6, WES was undertaken as described[27,28] using ANNOVAR and SAMtools. Publically available databases (1000 Genomes, ExAC, MitoCarta) and data from the Sequencing Initiative Suomi Project (SISu), an exome data collection of more than 6,000 Finnish individuals, were used for further variant filtering alongside in-house exome repositories.

Construction of Lactococcal Expression Strains and Site-Directed Mutagenesis

Codon-optimized *SLC25A4* was synthesized by GenScript. Site-directed mutations were introduced by overlap-extension PCR[29] with KOD HotStart polymerase (Novagen). Wild-type and mutant genes were cloned into the expression vector pNZ8048 under the control of a nisin A-inducible promoter.[30,31] The plasmid was transformed in *L. lactis* strain NZ9000 by electroporation, as previously described.[31] Vectors were isolated by miniprep (QIAGEN), according to the manufacturer's instructions with one alteration; 10 mg/mL lysozyme was added to the lysis buffer and the resuspended cells were incubated at 55°C for 20 min prior to lysis; Sanger sequencing confirmed successful transformants.

Growth of *Lactococcus lactis* and Membrane Isolation

Pre-cultures of *L. lactis* were obtained by inoculating M17 medium supplemented with 1% (w/v) glucose and 5 μg/mL chloramphenicol from glycerol stocks and incubating the cultures overnight at 30°C with no aeration. The OD_{600} was measured and the cells diluted to a starting OD_{600} of 0.1 in fresh M17 medium supplemented with 1% (w/v) glucose and 5 μg/mL chloramphenicol. Cells were grown at 30°C with no aeration until the OD_{600} reached 0.5; the expression of the recombinant proteins was induced by addition of nisin A with a dilution of 1:10,000 of spent M17 medium from nisin A excreting *L. lactis* strain NZ9700. The cells were grown for a further 140 min at 30°C, harvested by centrifugation (6,000 × *g*, 15 min, 4°C), resuspended in PIPES buffer (10 mM PIPES [pH 7.0], 50 mM NaCl), and collected by centrifugation.

Sample Preparation, SDS-PAGE, and Western Blot Analysis

Human fibroblasts were trypsinized, pelleted, and resuspended in cell lysis buffer (50 mM Tris-HCl [pH 7.5], 130 mM NaCl, 2 mM $MgCl_2$, 1% Nonidet P-40, 1 mM phenylmethylsulfonyl fluoride [PMSF], and protease inhibitor cocktail [Pierce]). Cell lysates were vortexed briefly and centrifuged at 500 × *g* for 5 min at 4°C and the supernatant was retained. Skeletal muscle samples were powdered in liquid nitrogen using pestle and mortar. 1 mL of RIPA buffer (1% Igepal, 1.5% Triton X-100, 0.5% sodium deoxycholate, 0.1% sodium dodecyl sulfate [SDS], 10 mM β-mercaptoethanol, 1 mM PMSF, and 1× EDTA free protease inhibitor cocktail [Pierce]) was added to powdered tissue and incubated on ice for 45 min. After 2 × 15 s homogenization with a polytron homogenizer, muscle lysates were cleared by centrifugation at 14,000 × *g* for 10 min at 4°C and supernatant retained. Cell and muscle lysates were incubated with sample dissociation buffer (final concentrations: 6.25 mM Tris-HCl [pH 6.8], 2% SDS, 10% glycerol, 0.01% bromophenol blue, and 100 mM DTT) for 30 min at 37°C, separated by 12% SDS-PAGE and immobilized by wet transfer (100 V, 1 hr at 4°C) on to PVDF membrane (Immobilon-P, Millipore) in 25 mM Tris, 192 mM glycine, 0.02% SDS, and 15% methanol. Proteins of interest were bound by overnight incubation at 4°C with antibodies against COXI (Abcam cat# ab14705, RRID: AB_2084810), SDHA (Abcam cat# ab14715, RRID: AB_301433), Porin/VDAC1 (Abcam cat# ab14734, RRID: AB_443084), UQCRC2 (Abcam cat# ab14745, RRID: AB_2213640), NDUFB8 (Abcam cat# ab110242, RRID: AB_10859122), ATP5A (Abcam cat# ab14748, RRID: AB_301447), OXA1L (Proteintech cat# 21055-1-AP, RRID: AB_10695769), ANT1 (Abcam cat# ab110322, RRID: AB_10862212), and TIMM22 (Abcam cat# ab167423) followed by HRP-conjugated secondary antibodies (Dako Cytomation) and visualized using ECL-prime (GE Healthcare) and BioRad ChemiDoc MP with Image Lab software.

For analysis of membrane fractions from *L. lactis*, membrane proteins were separated by SDS-PAGE and stained by Instant Blue. For western blotting, proteins were transferred to PVDF membranes and probed with an anti-AAC antibody produced in chicken at 1:20,000 dilution for 1 hr, followed by an anti-chicken-HRP conjugate at 1:20,000 dilution for a further hour. The membrane was developed using Amersham ECL western blotting detection system for 30 min.

Transport Assays

Transport assays were carried out using a Hamilton MicroLab Star robot (Hamilton Robotics Ltd). Transport of ^{14}C-labeled ADP (Perkin Elmer) was initiated by the addition of 100 μL PIPES buffer with 1.5 μM ^{14}C-ADP to approximately 200 μg whole cells in a MultiScreenHTS-HA 96-well filter plate (pore size = 1 μm; Millipore). The transport was stopped at 0 s, 20 s, 40 s, 60 s, 90 s, 120 s, 300 s, 600 s, 900 s, 10 min, 20 min, and 30 min by the addition of 200 μL ice-cold PIPES buffer and rapid filtration, followed by two additional wash steps with 200 μL ice-cold PIPES buffer. Levels of radioactivity in the vesicles were measured by adding 200 μL MicroScint-20 (Perkin Elmer) and by quantifying the amount of radioactivity with the TopCount scintillation counter (Perkin Elmer). Initial rates were determined by linear regression.

Yeast Strains, Plasmids, and Media

Yeast strains used were W303-1B (*MATα ade2-1 leu2-3,112 ura3-1 his3-22,15 trp1-1 can1-100 AAC1 AAC2*)[32] and its isogenic *aac1 aac2* mutant WB12 (*MATα ade2-1 trp1-1, ura3-1 can1-100 aac1:: LEU2 aac2:: HIS3*).[33]

Strains were grown in SC synthetic defined media (0.19% YNB without amino acids and NH_4SO_4 powder (Formedium), 0.5% NH_4SO_4) supplemented with 1 gr/L dropout mix without uracil.[34] Media were supplemented with various carbon sources at 2% (w/v) (Carlo Erba Reagents), in liquid phase or after solidification with 20 g/L agar (Formedium).

The *aac2*^{Arg96His} and *aac2*^{Arg252Gly} mutant alleles were obtained by PCR overlap technique with appropriate primers (details available on request) using the pFL38-*AAC2* as template.[35] The obtained fragment was cloned into the SalI-BamHI-digested pFL38.[36] WB12 or W303-1B were transformed with pFL38-*AAC2*, pFL38-*aac2*^{Arg96His}, pFL38-*aac2*^{Arg252Gly}, or the empty vector pFL38 through the Li-Ac method.[37]

Growth of *S. cerevisiae*, Membrane Isolation, and Fusions

Pre-cultures of *S. cerevisiae* were obtained by inoculating SC without uracil + 2% glucose medium from glycerol stocks and incubating the cultures overnight at 30°C with shaking at 225 rpm. The OD_{600} was measured and the cells diluted to a starting OD_{600} of 0.1 in fresh SC without uracil + 2% glucose medium. Cells were grown at 30°C with shaking at 225 rpm for 20 hr. Cells were harvested by centrifugation (6,000 × *g*, 15 min, 4°C), washed in ice-cold MilliQ, and centrifuged as before. The cells were resuspended in breaking buffer (650 mM sorbitol, 100 mM Tris-HCl [pH 8.0], 5 mM EDTA, 5 mM amino hexanoic acid, 5 mM benzamidine, 0.2% BSA) and disrupted mechanically with a cell disruptor (Constant Systems) at 33,000 psi. Whole cells and debris were removed by centrifugation (10,800 × *g*, 15 min, 4°C), and the membranes were collected by ultracentrifugation (138,000 × *g*, 1 hr, 4°C). Pellets were resuspended in washing buffer (650 mM sorbitol, 100 mM Tris-HCl [pH 8.0], 5 mM amino hexanoic acid, 5 mM benzamidine) and collected by ultracentrifugation as before. Pellets were finally resuspended in PIPES buffer (10 mM PIPES [pH 7.0], 50 mM NaCl) to a total protein concentration of approximately 3 mg/mL and stored in liquid nitrogen.

To make membrane fusions, 1 mg yeast membranes were mixed with 5 mg 3:1 *E. coli* polar lipid extract and egg yolk phosphatidylcholine (Avanti Polar Lipids) liposomes, diluted to a final volume of 900 μL with PIPES, and fused by seven cycles of freezing in liquid nitrogen and thawing at room temperature before storage in liquid nitrogen. The membrane vesicle fusions were thawed and 100 μL 50 mM ADP added. Vesicles were extruded 11 times through a 1 μm pore size polycarbonate filter, passed

through a pre-equilibrated PD10 column to remove external substrate, collected in 1.6 mL PIPES buffer, and diluted 4-fold before use. Transport experiments were carried out as described above.

Drop Tests

Pre-cultures of *S. cerevisiae* were obtained by inoculating SC without uracil + 2% glucose medium from glycerol stocks and incubating the cultures overnight at 28°C with shaking at 225 rpm. Cells were collected by centrifugation (3,000 rpm, 2 min, room temperature), washed twice with MilliQ, and diluted to a starting OD_{600} of 1.0. A serial 10-fold dilution was set up in sterile MilliQ, and 5 μL of each dilution was dispensed onto SC plates without uracil, supplemented with either 2% glucose, 2% ethanol, or 2% glycerol. The plates were incubated at 28°C or 37°C for 72 hr.

Yeast Analysis

Respiratory activity was performed as previously described.[38,39] Reduced versus oxidized cytochrome spectra of yeast cells cultured for 24 hr at 37°C in SC without uracil, supplemented with glucose at non-repressing concentration of 0.6% were recorded (Varian Cary300 UV-VIS Spectrophotometer) at room temperature.

Results

Case Reports

Subject 1

This female infant was born at full term via Caesarean section for a breech presentation to non-consanguineous white British parents with a healthy older sister. She required ventilation at birth and was noted to have elevated lactate levels in blood shortly after birth, requiring correction with sodium bicarbonate. Plasma lactate levels ranged from 3 to 15 mmol/L (normal range, 0.7–2.1 mmol/L), and her CSF lactate was also elevated. Echocardiography showed mild left ventricular hypertrophy and urine organic acid testing detected low levels of ethylmalonic acid. Although she was profoundly hypotonic, her cranial MRI was reported as normal for age. Despite multiple attempts to extubate she did not demonstrate any improvement in respiratory drive and after obtaining a diagnostic muscle biopsy, care was withdrawn at 14 days. The parents have since had a healthy son (Figure 1).

Subject 2

This male infant was born to non-consanguineous white British parents after an unremarkable pregnancy. Labor was induced at 42 weeks and he was delivered by ventouse extraction weighing 4,260 g. Apgar scores were 10 and 10 at 1 and 5 min, respectively, but he developed tachypnoea and signs of respiratory distress at 10 hr, requiring intubation and mechanical ventilation. Serial blood gases revealed profound and persistent lactic acidosis (peak lactate of 14 mmol/L) with concomitant elevation of CSF lactate (3.3 mmol/L). Blood cultures identified *Enterococcus faecalis* and he received treatment with intravenous antibiotics. Echocardiogram and electroencephalogram were normal, but cranial MRI scan showed a small infarct in the right thalamus. Skeletal muscle biopsy revealed evidence of mitochondrial disease (Table 1). There was no improvement in the lactic acidosis despite antibiotic, biotin, thiamine, and ubiquinone treatment. Attempts to extubate were unsuccessful on several occasions and the family agreed to a palliative care approach. Discharge to a hospice for terminal care took place 9 days after birth.

Subject 3

This female infant was born at term weighing 3,520 g and was the second child of healthy non-consanguineous Estonian parents. She was floppy at birth with paucity of limb movements and hyporeflexia. She required low-level supplemental oxygen in the immediate postnatal period, but was breathing air from 5 to 48 hr of age, at which point she deteriorated with a decrease in conscious level, feeding difficulties, and respiratory impairment. She was intubated and mechanically ventilated. Elevated lactate was noted in blood (9.7 mmol/L, normal < 2.2 mmol/L) and cerebrospinal fluid (4.8 mmol/L, normal range 1.1–2.4 mmol/L). Urine analysis revealed increased excretion of lactate, 2-hydroxybutyric acid, fumarate, ketoglutarate, ethylmalonate, and 4-hydroxyphenyl lactate. Echocardiography showed a mildly enlarged apex of the left ventricle. At day 7, despite treatment with coenzyme Q_{10} (20 mg/kg/day), riboflavin (100 mg/kg/day), creatine (200 mg/kg/day), and L-carnitine (100 mg/kg/day), she developed persistent generalized clonic seizures without electroencephalographic (EEG) abnormalities. Serial MRIs initially demonstrated communicating hydrocephalus and subsequently progressive cerebral white matter atrophy. Presently, at 6 years, she has a tracheostomy, is unable to speak, and can breathe independently for less than 1 hr. She is reliant on a wheelchair due to proximal muscle weakness, but can walk briefly with a frame. Communication is by pointing or pictogram.

Subject 4

This female was born to Ashkenazi Jewish parents after an IVF pregnancy that was remarkable for intrauterine growth retardation, cardiac echogenic focus, and enlarged renal pelvis on fetal ultrasound. She was delivered at 28 weeks gestation by emergency cesarean section due to maternal HELLP (hemolysis, elevated liver enzymes, and low platelets) syndrome, weighed 920 g, and had Apgar scores of 5 and 8 at 1 and 5 min, respectively. She required intubation and ventilation for respiratory distress syndrome and required dopamine for hypotension. A patent ductus arteriosis (PDA) was treated with ibuprofen. Brain ultrasound revealed a small asymmetric, predominantly left-sided, grade II intraventricular hemorrhage, though subsequent cranial MRI was normal. Bronchoscopy and chest CT were normal but she became ventilator dependent. Cardiac echo revealed hypertrophic cardiomyopathy that was resolved after steroid therapy was stopped. Tracheostomy was performed at 5 months and a gastrostomy with fundoplication at 6 months. At the age of 7 months she was hypomimic, dysphagic, and profoundly hypotonic. She had tongue fasciculation, was areflexic, and had no antigravity limb movements, but electromyography showed no evidence of myopathy or denervation, though

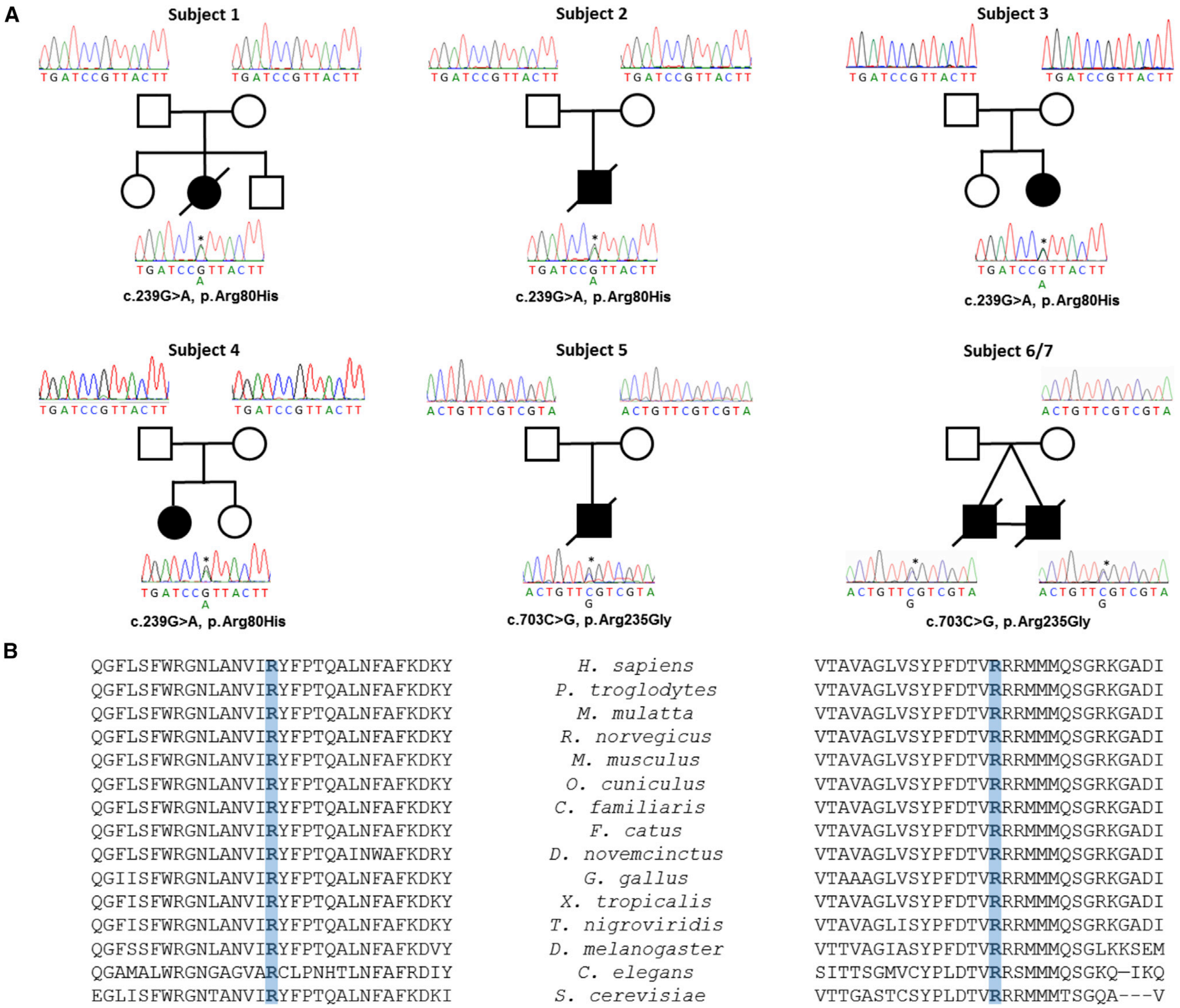

Figure 1. The c.239G>A (p.Arg80His) and c.703C>G (p.Arg235Gly) *SLC25A4* Mutations Are De Novo Affecting Evolutionary Conserved Residues

(A) Pedigrees of six families showing each clinically affected subject and confirmatory sequencing chromatograms to show that the c.239G>A (p.Arg80His) or c.703C>G (p.Arg235Gly) heterozygous variants in each affected individual are not present in respective parental samples, indicating de novo occurrence.

(B) Multiple sequence alignment of this region of the AAC1 amino acid sequence (GenBank: NP_001142.2) was performed using ClustalOmega and confirms that the p.Arg80His (left) and p.Arg235Gly (right) alterations affect evolutionarily conserved residues (shaded blue).

single-fiber results suggested a possible congenital myasthenia. Treatment with mestinone, ephedrine, and albuterol did not improve her condition. Urinary Krebs cycle metabolites included fumarate, alpha-ketoglutarate, and succinate. Marked dicarboxylic acids were also identified, with increased glutaric acid, 2-hydroxyglutaric, ethylmalonic, and methylsuccinic acid. The child developed atopic dermatitis and severe allergy to sesame and peanuts. Auditory brain stem response showed mild conductive hearing impairment. Ophthalmologic examination was normal. At present, aged 4 years, she has normal cognition and is fed via gastrostomy, requiring ventilation 24 hr a day. She remains markedly hypotonic and weak, being unable to crawl or stand. She has very limited verbal communication but receptive language is good and she communicates with a computerized aid. The parents have since had a healthy daughter, who was naturally conceived.

Subject 5

This male infant was born to Ashkenazi Jewish parents at 39 weeks gestation after a normal pregnancy and weighed 3,250 g. He had Apgar scores of 1, 4, and 7 at 1, 5, and 10 min, respectively, and required suction of meconium from his airways, intubation, and mechanical ventilation. On admission to the neonatal intensive care unit, he was noted to be profoundly hypotonic with severe head lag and had bilateral abnormalities of his wrist joints.

Table 1. Biochemical and Clinical Findings in Individuals with De Novo *SLC25A4* Mutations

Subject Details		*SLC25A4* Variant	Assessment of Skeletal Muscle						Clinical Features		
**ID**	**Sex**	**cDNA (NM_001151.3), Protein (NP_001142.2)**	**Muscle Biopsy Findings**	**RCC**	**% Mean Enzyme Activity**	**Absolute Values**	**Control Mean (Reference Range)**	**mtDNA Copy Number**	**Age of Onset**	**Clinical Course**	**Other Clinical Features and Relevant Family History**
1	female	c.239G>A (p.Arg80His)	global COX deficiency; excessive lipid accumulation	I	5% (↓↓↓)	0.005	0.104 ± 0.036 (n = 25)	<5%	birth	died 14 d	ventilator dependent from birth, hypotonia, raised CSF and serum lactate, hypertrophic cardiomyopathy
				II	172%	0.25	0.145 ± 0.047 (n = 25)				
				III	16% (↓↓)	0.089	0.554 ± 0.345 (n = 25)				
				IV	9% (↓↓↓)	0.098	1.124 ± 0.511 (n = 25)				
2	male	c.239G>A (p.Arg80His)	global COX deficiency; occasional fibers showing enhanced mitochondrial activity; excessive lipid accumulation	I	17% (↓↓)	0.018	0.104 ± 0.036 (n = 25)	<5%	birth	died 9 d	ventilator dependent, raised CSF and serum lactate, encephalopathy, seizures
				II	177%	0.256	0.145 ± 0.047 (n = 25)				
				III	20% (↓↓)	0.112	0.554 ± 0.345 (n = 25)				
				IV	14% (↓↓)	0.16	1.124 ± 0.511 (n = 25)				
3	female	c.239G>A (p.Arg80His)	excessive lipid accumulation on histology and EM; mitochondria showing disorganization of cristae structure	I	18% (↓↓)	28	154 ± 59 (n = 8)	≈34%	birth	alive 6 y	ventilator dependent, paucity of limb movements, raised CSF and serum lactate, seizures, wheelchair bound, tracheostomized
				II+III	46% (↓)	51	110 ± 70 (n = 8)				
				IV	5% (↓↓↓)	58	1150 ± 400 (n = 8)				
4	female	c.239G>A (p.Arg80His)	ND	I	ND	ND	ND	ND	birth	alive 4 y	ventilator dependent from birth, tracheostomized, paucity of limb movements
				II							
				III							
				IV							
5	male	c.703C>G (p.Arg235Gly)	global COX deficiency	I	12% (↓↓)	0.014	0.116 ± 0.018 (n = 40)	20%	birth	died 3 m	ventilator dependent, paucity of limb movements, lactic acidosis, concentric hypertrophy, tracheostomized, died after cardiac arrest
				II	116%	0.108	0.097 ± 0.015 (n = 40)				
				II+III	1% (↓↓↓)	0.014	0.142 ± 0.039 (n = 40)				
				IV	5% (↓↓↓)	0.033	0.664 ± 0.113 (n = 40)				
6	male	c.703C>G, (p.Arg235Gly)	global COX deficiency	I+III	109%	153	140 ± 70 (n = 7)	ND	birth	died 1.5 m	identical twin brother of subject 7, ventilator dependent from birth, raised CSF and serum lactate
				II	57%	93	162 ± 57 (n = 7)				
				II+III	25% (↓↓)	49	197 ± 88 (n = 7)				
				IV	3% (↓↓↓)	88	2521 ± 759 (n = 7)				
7	male	c.703C>G (p.Arg235Gly)	ND	I	ND	ND	ND	11%–13%	birth	died 1.5 m	identical twin brother of subject 6, lack of respiratory drive from birth, ventilator dependent, raised CSF and serum lactate
				II							
				III							
				IV							

Respiratory chain complex (RCC) activities are expressed as nmols of substrate.min^{-1}.unit citrate $synthase^{-1}$. ↓, ≤50% control enzyme activity; ↓↓, ≤25% control enzyme activity; ↓↓↓, ≤10% control enzyme activity.

Respiration deteriorated, primary pulmonary hypertension of the newborn (PPHTN) was suspected, and treatment with nitric oxide (up to 40 ppm) and continuous inotropic therapy was initiated. Despite aggressive management, echocardiogram performed a few hours later demonstrated severe PPHTN, though this did improve over the course of the following 2 weeks. Attempted extubation at this point was, however, unsuccessful; he developed paradoxical breathing and bradycardia. Neurological status deteriorated over the following week, with increasing hypotonia, paucity of limb movement, generalized weakness, hypomimia, tongue fasciculation, dysphagia, and apnea. He was noted to be areflexic and a diagnosis of spinal muscular atrophy was considered. Laboratory investigations revealed elevated blood lactate (15 mmol/L; normal range 0.9–2 mmol/L) and hyperammonemia (300 mcg/dL; normal < 50 mcg/dL) that subsequently normalized. Blood amino acids and urinary amino and organic acids were normal. Repeat echocardiogram revealed concentric hypertrophy and although cranial MRI demonstrated a normal brain structure, there were small subacute infarcts in the splenium of the corpus callosum and right thalamus consistent with hypoxic ischemic encephalopathy. Tracheostomy and a feeding jejunostomy were inserted but at the age of 3 months, after a gradual worsening in cardiac hypertrophy and cardiac function, the child suffered a fatal cardiac arrest.

Subject 6

This identical twin brother of subject 7 was born to non-consanguineous Finnish parents. The twin pregnancy was normal until 33 weeks when there was a spontaneous rupture of membranes. He was delivered by Ventouse extraction weighing 1,855 g with Apgar scores of 8 and 7 at 1 and 5 min, respectively. He was hypotonic and had breathing difficulties from birth that progressed quickly to apnea and dependence on mechanical ventilation. His respiratory drive showed no improvement and he died at 6 weeks of age. Laboratory investigations showed elevated lactate in both blood (5.2 mmol/L; normal range 0.7–1.8 mmol/L) and CSF (3.9 mmol/L; normal range 0.6–2.7 mmol/L). Brain MRI, EEG, ophthalmological examination, glucose, creatine kinase, ammonia, plasma amino acids, urine organic acids, and phytanic acid were all normal.

Subject 7

This identical twin brother of subject 6 was born by emergency Cesarean section due to asphyxia with a birth weight of 1,850 g and Apgar scores of 1, 2, and 3 at 1, 5, and 10 min, respectively. He had hypoxic ischemic encephalopathy (HIE), was apnoeic, and needed resuscitation. He was hypotonic, had prolonged hypoventilation, and was respirator dependent with no spontaneous breathing and few spontaneous movements. As with his brother, spontaneous respiration did not improve and he died at 1.5 months of age. EEG was initially abnormal due to HIE, but normalized within 2 weeks. Mild periventricular hemorrhage was seen on cranial MRI. Lactate was elevated in blood (5.4 mmol/L; normal range 0.7–1.8 mmol/L) and CSF (4.2 mmol/L; normal range 0.6–2.7 mmol/L) and excretion in urine was also elevated. Cardiac and abdominal ultrasound, ophthalmological examination, electromyography, ammonia, urine metabolic screening, and plasma amino acids were normal.

Diagnostic Mitochondrial Investigations of Subject Muscle

Five of the seven subjects within our cohort (subjects 1, 2, 3, 5, and 6) had diagnostic muscle biopsies for the evaluation of suspected mitochondrial disease using a range of histopathological and biochemical assays (Table 1). Cytochrome *c* oxidase (COX) oxidative enzyme histochemistry in muscle from subjects 1 and 2 revealed a generalized loss of enzyme activity (Figure S1); the SDH reaction in subject 2 showed fibers with enhanced mitochondrial activity while Oil Red O (subject 1) or Sudan Black (subject 2) staining confirmed prominent lipid accumulation (Figure S1). Histopathological assessment of subject 3's muscle biopsy revealed small and rounded muscle fibers with little variation in fiber size and without evidence of atrophy, necrosis, fibrosis, or inflammatory changes; Oil Red O staining showed lipid deposition (Figure S1) and modified Gomori trichrome staining disclosed abnormal staining around the subsarcolemmal region of some fibers; unfortunately, histochemical assessment of oxidative enzyme activities was not undertaken. Electron microscopy investigation confirmed the accumulation of both lipid and glycogen, revealing numerous mitochondria with disorganized cristae (Figure S1), although paracrystalline inclusions were not detected.

The assessment of mitochondrial respiratory chain complex activities in subjects 1, 2, and 5 showed decreased activities of complexes I, III, and IV, with a compensatory increase in the activity of complex II (Table 1). Subject 3 showed low complex I and IV activities in muscle, while the combined measurement of complexes II+III (succinate: cytochrome *c* reductase activity) was within the normal range. Subject 6 also had impaired complex IV activity and whereas the combined measurement of complexes II+III were also low, the combined measurement of complexes I+III was within the normal range (Table 1). These defects in OXPHOS enzyme activities were associated with mtDNA depletion in subject muscle; qPCR revealed a striking loss of mtDNA copy number to <5% of control levels in both subjects 1 and 2, whereas subjects 3, 5, and 7 demonstrated decreased levels between 11%–34% of control levels (Table 1).

Molecular Genetic Investigations

The demonstration of severe mtDNA depletion prompted screening of genes associated with mtDNA depletion myopathy (*TK2* and *RRM2B*) in subjects 1 and 2 prior to WES. In subject 3, mutations in *POLG* and *TK2* were excluded after chromosomal microarray analysis revealed no abnormalities. Subjects 6 and 7 were previously

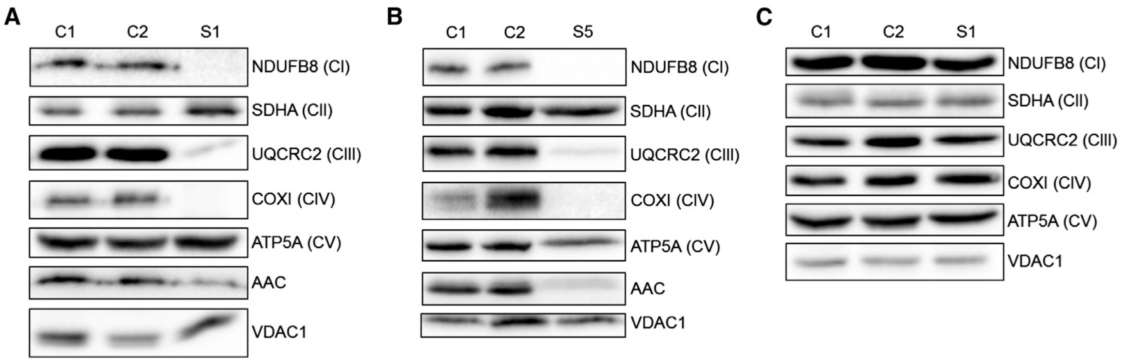

Figure 2. The p.Arg80His and p.Arg235Gly Alterations Cause Decreased AAC1 Levels and Loss of OXPHOS Subunits Specifically in Skeletal Muscle
(A) Western blot analysis of OXPHOS complex subunits and AAC (Abcam cat# ab110322, RRID: AB_10862212) performed on skeletal muscle lysates from subject 1 (p.Arg80His).
(B) Western blot analysis of OXPHOS complex subunits and AAC (all isoforms) performed on skeletal muscle samples from subject 5 (p.Arg235Gly).
(C) Western blot analysis of OXPHOS complex subunits performed on fibroblast lysates from subject 1.

screened for mutations in mtDNA, *SCO2*, *TK2*, and *DGUOK*.

WES data from subject 1 was reported in a large study of individuals with multiple mitochondrial respiratory chain deficiencies (patient number 43),[3] identifying several undocumented heterozygous variants in mitochondrially targeted candidates. Subsequent Sanger sequencing of parental samples revealed that the heterozygous c.239G>A (p.Arg80His) *SLC25A4* (GenBank: NM_001151.3; ClinVar: SCV000297809) variant was de novo, later confirmed by microsatellite analysis to firmly establish the parentage of subject 1 (data not shown). WES of subjects 2, 3, and 4—undertaken at different mitochondrial diagnostic centers—identified the identical heterozygous c.239G>A (p.Arg80His) *SLC25A4* mutation. Given the similarity in clinical presentation, it was deemed highly likely that this de novo variant was pathogenic; indeed, for subject 2, WES was undertaken for the family trio, with the absence of the mutation in all parental samples designating a de novo origin. Sanger sequencing of parental samples firmly established that the c.293G>A mutation had arisen independently in each of the four case subjects (Figure 1A).

WES of subject 5 identified a different heterozygous *SLC25A4* mutation, c.703C>G (p.Arg235Gly). The similarity in clinical features between subject 5 and subjects 1–4 suggested that this missense mutation was likely disease causing. Subsequently, we became aware of twins (subjects 6 and 7) that had a very similar clinical course to the other affected individuals and had remained without a genetic diagnosis after WES. Revisiting these data facilitated the identification of an identical c.703C>G (p.Arg235Gly) *SLC25A4* mutation in both individuals, which had previously not been considered during variant filtering based on a presumed autosomal-recessive cause of their disease phenotype. Sanger sequencing confirmed the presence and de novo nature of the c.703C>G (p.Arg235Gly) mutation in subject 5. In the case of subjects 6 and 7, Sanger sequencing confirmed the presence of the c.703C>G (p.Arg235Gly) mutation in each affected individual and the absence of the mutation in the mother, but unfortunately the father's DNA sample was not available for genetic analysis.

The de novo c.239G>A (p.Arg80His) mutation is predicted to be pathogenic based on various in silico prediction tools including PolyPhen-2 (HumDiv score 0.966),[40] SIFT (score 0),[41] and CADD (phred score 34).[42] The amino acid position is highly conserved across different species (Figure 1B) (PhyloP score 6.072). The c.703C>G (p.Arg235Gly) mutation is also predicted to be pathogenic based on PolyPhen-2 (HumDiv score 0.883),[40] SIFT (score 0),[41] and align GVGD (Class C65, GV: 0.00, GD: 125.13)[43] and the amino acid is notably conserved throughout evolution (Figure 1B).

Steady-State Levels of Respiratory Chain Components and AAC1 Protein

Western blot analysis of muscle lysate from subject 1 (p.Arg80His) showed a decrease in the steady-state levels of OXPHOS components of complex I (NDUFB8), complex III (UQCRC2), and complex IV (COXI) with an increase in SDHA (complex II) expression (Figure 2A). A similar OXPHOS defect was seen in skeletal muscle from subject 5 (p.Arg235Gly) (Figure 2B). These data corroborate the pattern of respiratory chain complex deficiencies observed by direct enzyme assay (Table 1). Furthermore, skeletal muscle from each affected individual clearly showed decreased steady-state levels of AAC1, confirming a functional consequence of both de novo *SLC25A4* mutations (Figures 2A and 2B). The expression of ADP/ATP carrier isoforms differs between tissues. Skin cells predominantly express AAC2 (also known as ANT2, encoded by *SLC25A5* [MIM: 300150]) and very little AAC1.[19] Consequently, subject fibroblasts did not express the OXPHOS defect observed in muscle as analyzed by western blot (Figure 2C).

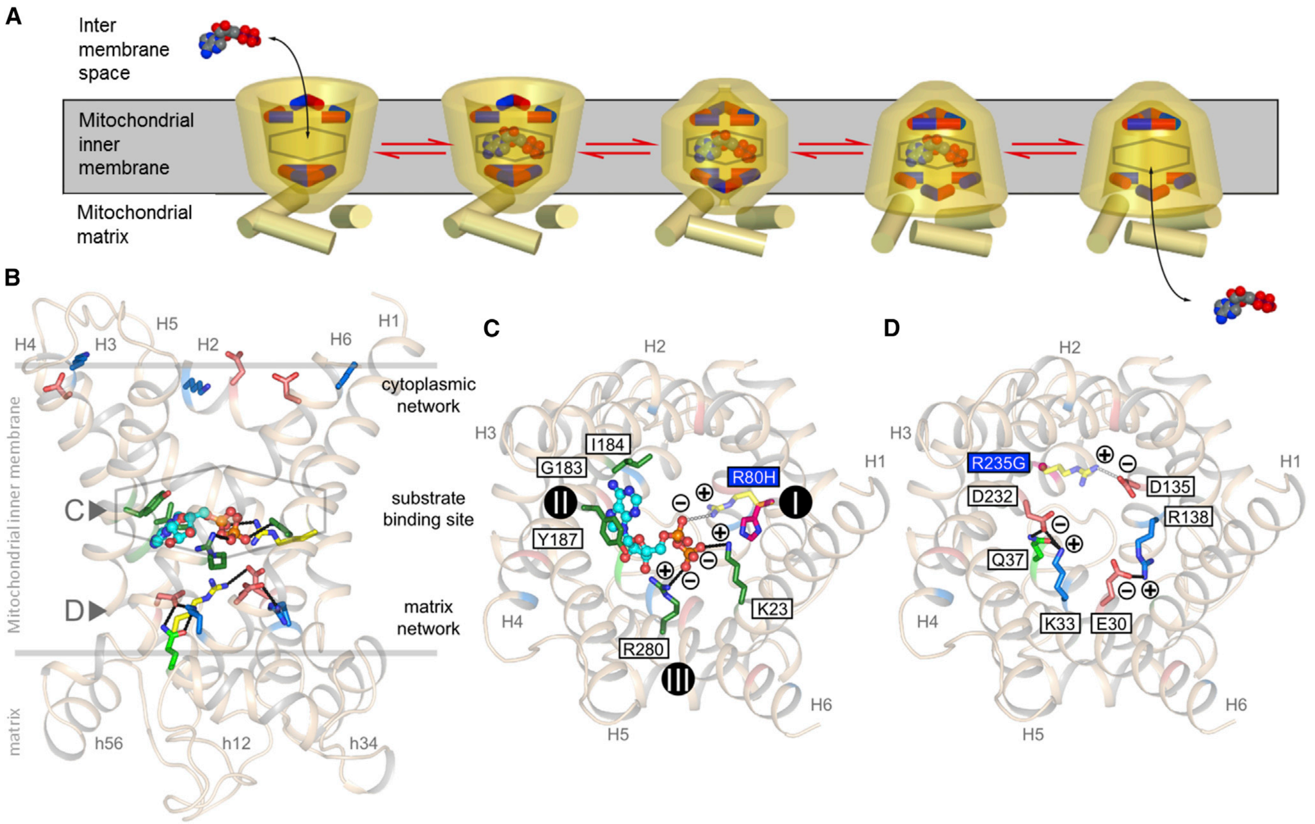

Figure 3. The Functional Elements of the Human Mitochondrial ADP/ATP Carrier
(A) Transport cycle of the ADP/ATP carrier. Disruption and formation of the cytoplasmic and matrix salt bridge networks (top and bottom, respectively) change the accessibility of the central substrate binding site (hexagon) to either side of the membrane.[44,47]
(B) Lateral view of the human ADP/ATP carrier from the membrane, showing the residues of the matrix and cytoplasmic networks (blue and red sticks) and substrate binding site (green sticks, hexagon). ADP (light blue ball and stick) and the glutamine brace (light green stick)[48] are also shown. The residues that are mutated are shown in yellow.
(C) Cytoplasmic view of the carrier showing only the residues of the proposed substrate-binding site. The residues of the p.Arg80His (R80H) mutation are shown in yellow and magenta, respectively. The contact points of the substrate binding site are indicated by roman numerals.[50]
(D) Cytoplasmic view of the carrier showing only the residues of the matrix salt bridge network. The residues of the p.Arg235Gly (R235G) mutation are shown in yellow and magenta, respectively. The comparative model of human AAC1 is based on the structure of the bovine carrier[46] and was extended at the C terminus. The ionic interactions are indicated with plus and minus signs.

Location of the Mutations in the Mitochondrial ADP/ATP Carrier

In the transport cycle, the mitochondrial ADP/ATP carrier cycles between two states: a matrix state in which the central substrate binding is accessible to the mitochondrial matrix and a cytoplasmic state in which the binding site is accessible to the intermembrane space, which is confluent with the cytosol[7,44] (Figure 3A). A three-fold salt bridge network on the matrix side[45,46] and on the cytoplasmic side of the carrier[44,47,48] are involved in opening and closing of the carrier to either side of the membrane in an alternating way, regulating access to the central substrate binding site (Figure 3A).[44] The human carrier is closely related to the bovine carrier, for which an atomic structure is available.[46] A comparative model of the human carrier was built to locate the position of the p.Arg80His and p.Arg235Gly alterations. The view from the membrane shows the location of the central substrate-binding site and the matrix and cytoplasmic salt bridge networks (Figure 3B). The main contact points involved in substrate binding, indicated by roman numerals I, II, and III, are located on the even-numbered α helices.[49,50] The proposed substrate binding site of the human mitochondrial ADP/ATP carrier consists of Gly183, Ile184, and Tyr187 (contact point II) for binding of the adenine moiety and Arg80 (contact point I), Lys23, and Arg280 (contact point III) for binding of the phosphate groups (Figure 3C).[49,50] The p.Arg80His alteration affects contact point I of the proposed substrate-binding site of AAC1 (Figure 3C). The histidine substitution may conserve the positive charge, depending on the local pH (pK_a 6.0), but would introduce a shorter and less flexible residue.

The matrix network has salt bridge interactions between residues Lys33 and Asp232, Arg235 and Asp135, and Arg138 and Glu30 (Figure 3D).[46] In addition, there is a glutamine brace interacting with Asp232-Lys33 residues.[48] The p.Arg235Gly alteration abolishes one of the

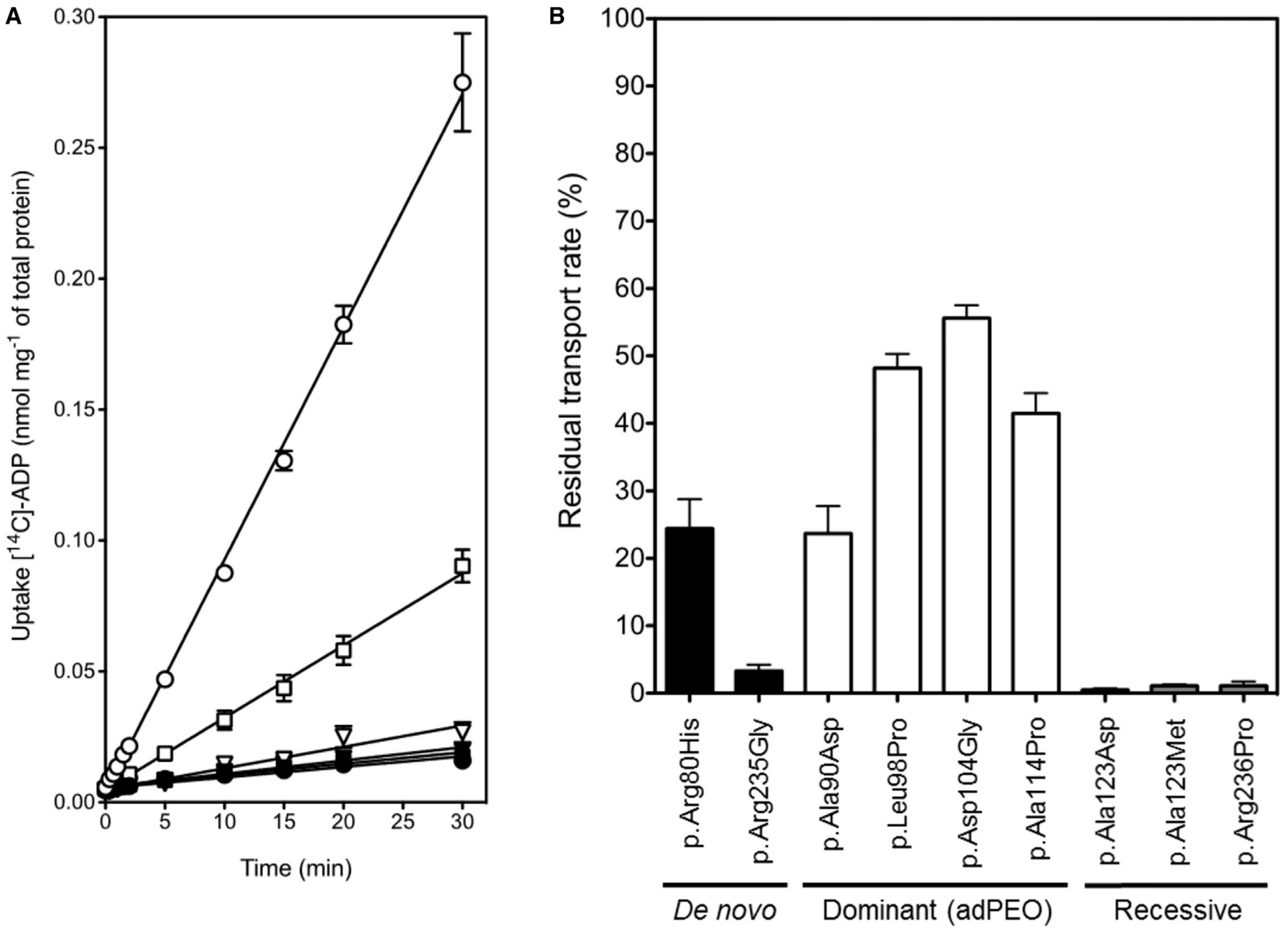

Figure 4. The p.Arg80His and p.Arg235Gly Alterations Impair ADP/ATP Transport

(A) ADP uptake curves of whole cells of *L. lactis*-expressing AAC1 (open circle), AAC1 p.Arg80His (open square), and AAC1 p.Arg235Gly (open triangle). To determine the non-specific binding, the specific inhibitor carboxyatractyloside was added (closed symbols). The error bars represent the standard deviation of eight assays.

(B) Residual transport activity relative to wild-type AAC1, corrected for background binding and differences in expression levels, as determined by the uptake rate of radiolabeled ADP into *L. lactis*-expressing human AAC1 with de novo mutations described here (black bars), adPEO mutations (white bars), or the recessive mitochondrial myopathy and cardiomyopathy mutations (gray bars). The data are represented by the average and standard deviation of eight transport assays.

three salt bridge interactions, which may affect the overall strength of the matrix network and function of the carrier.[51]

The p.Arg80His and p.Arg235Gly Alterations Impair the Function of the ADP/ATP Carrier

It has been shown that human AAC1 can be expressed in a functional form in the cytoplasmic membrane of the Gram-positive bacterium *Lactococcus lactis*.[52] To study the effect of the mutations on transport activity, the c.239G>A and c.703C>G mutations were introduced by site-directed mutagenesis into human *SLC25A4* in the expression vector pNZ8048. The transport activity of the carriers was determined in whole cells by monitoring the uptake of radiolabeled ADP in exchange for endogenous adenine nucleotides. To determine the non-specific background, the specific inhibitor carboxyatractyloside (CATR) was used in control experiments (Figure 4A). The uptake of ADP was severely reduced in the AAC1 that contained the disease mutations. Subsequently, mutants with the previously reported *SLC25A4* pathogenic mutations were made for direct comparison. All proteins expressed to approximately the same levels in the cytoplasmic membrane of *L. lactis*, as judged by western blotting (Figure S2). Initial uptake rates were determined and they were corrected for differences in expression levels (Figure S2) and for non-specific binding, using the CATR controls. For each mutant, the residual transport rate was calculated as a percentage of the wild-type AAC1 rate. Both the p.Arg80His and p.Arg235Gly alterations had severe effects on transport activity with residual transport rates of 24% and 3%, respectively (Figure 4B). The adPEO-linked mutations were generally milder with residual transport rates ranging from 24% to 56%, whereas mutations that are reported to cause autosomal-recessive disease inactivated transport fully (<1%) (Figure 4B).

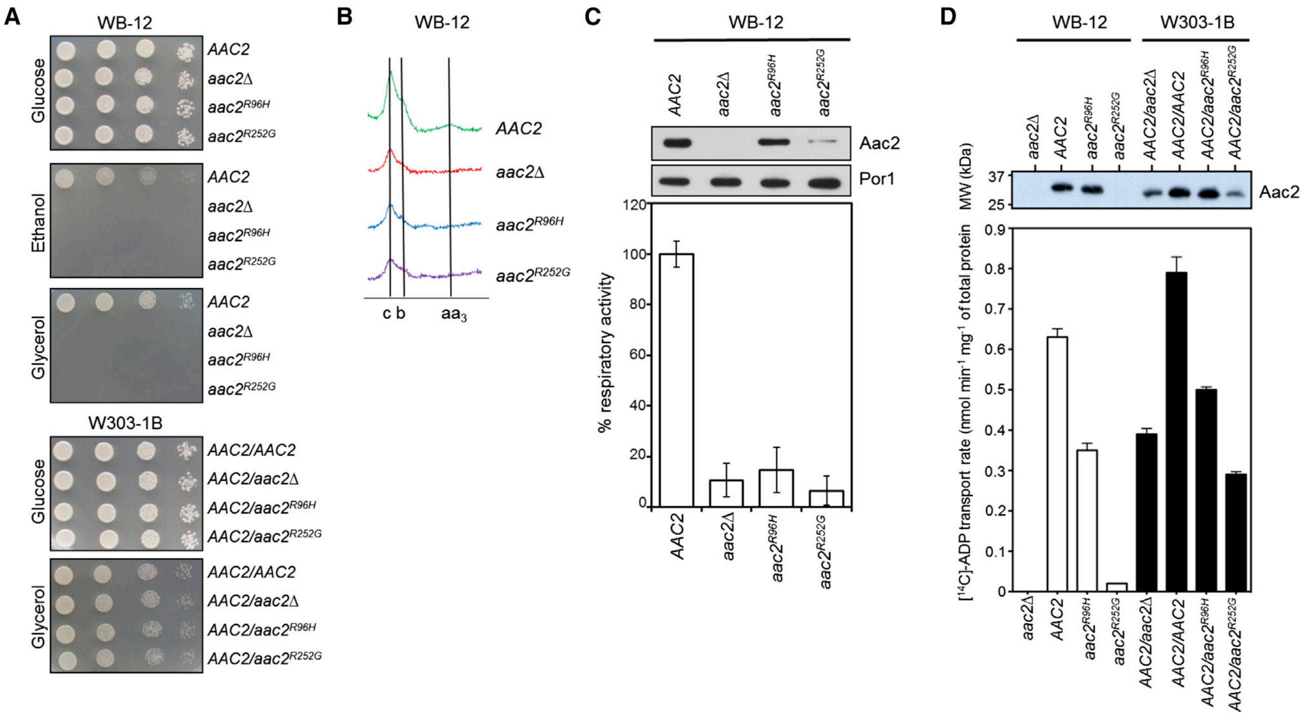

Figure 5. Phenotypic Analysis of the WB12 Strain Transformed with the Empty Vector, Wild-Type *AAC2*, *aac2*^{Arg96His}, or *aac2*^{Arg252Gly} Mutant Allele

(A) Assessment of an oxidative growth phenotype. Equal amounts of serial dilutions of cells from exponentially grown cultures (10^5, 10^4, 10^3, and 10^2 cells) were spotted onto SC plates without uracil, supplemented with either 2% glucose, 2% ethanol, or 2% glycerol. The growth was scored after 3 days of incubation at 28°C.

(B) Cytochrome profiles of cells grown in SC without uracil supplemented with 0.6% glucose at 37°C. The peaks at 550, 560, and 602 nm (vertical bars) correspond to cytochromes *c*, *b*, and aa_3, respectively. All the experiments were performed in triplicate.

(C) Respiratory activity (normalized to wild-type, performed in triplicate, error bars show standard deviation) and corresponding western blot analysis to show Aac2 expression.

(D) Transport activity as determined by the uptake rate of [^{14}C]-labeled ADP measured with fused mitochondrial membranes isolated from yeast strains and corresponding western blot analysis to illustrate appropriate Aac2 expression. Error bars describe the standard deviations of four assays.

Complementation and Transport Studies in Yeast *Saccharomyces cerevisiae*

To validate the pathogenic role of the AAC1 missense mutations in vivo, we established ad hoc recombinant systems in the budding yeast *Saccharomyces cerevisiae*, a facultative aerobic organism. *AAC2* in yeast encodes Aac2, which is the ortholog of human mitochondrial AAC1. Both Arg80 and Arg235 residues of human AAC1 are conserved, corresponding to Arg96 and Arg252, respectively, in yeast Aac2. We introduced the p.Arg96His and p.Arg252Gly amino acid substitutions in the yeast Aac2 protein by site-directed mutagenesis of a recombinant *AAC2* cloned in the centromeric pFL38 vector. Mutant proteins were expressed in the *S. cerevisiae* WB12 strain (*aac2*) for complementation studies, as this strain lacks functional ADP/ATP carriers, and in W303-1B strain (*AAC2*), which expresses one copy of *AAC2* on the genome, to evaluate the dominance/recessivity of the *aac2*^{Arg96His} and *aac2*^{Arg252Gly} mutations. The yeast strain W303-1B *AAC2* was transformed with a plasmid carrying either wild-type *AAC2* or the *aac2*^{Arg96His} or *aac2*^{Arg252Gly} mutant alleles. The resulting *AAC2/AAC2* homoallelic and *AAC2/aac2*^{Arg96His} or *AAC2/aac2*^{Arg252Gly} heteroallelic strains model the diploid genetic organization of human cells.

Growth of yeast on either ethanol or glycerol as non-fermentable carbon sources is critically dependent on a functional ADP/ATP carrier and electron transport chain. Thus, yeast transformants were analyzed first for growth on plates containing non-fermentable carbon sources or glucose as a control. Plates were incubated at two different temperatures: 28°C, which is the optimal temperature for yeast, and 37°C, under temperature-induced stress conditions. Neither the *aac2*^{Arg96His} nor *aac2*^{Arg252Gly} mutant strains were able to grow on ethanol or glycerol at either temperature (Figure 5A), indicating that the mutant alleles are not able to complement the OXPHOS defect of the *aac2*-null mutant. The levels of expressed Arg96His mutant Aac2 were similar to wild-type (Figure 5C), so these data suggest that in the *aac2*^{Arg96His} strain, the transport rate of the mutant carrier is insufficient in vivo to sustain growth. In contrast, the p.Arg252Gly mutant Aac2 protein was expressed at very low levels in the *aac2*^{Arg252Gly} strain (Figure 5C), which may also account for the lack of growth on glycerol. All strains with the W303-1B background grew on glycerol, showing that the activity of Aac2 expressed

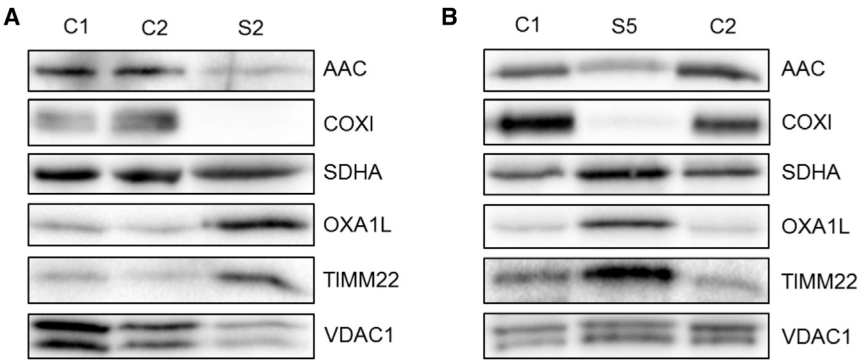

Figure 6. De Novo *SLC25A4* Mutations Do Not Decrease Mitochondrial Protein Import
(A) Western blot analysis of inner mitochondrial membrane proteins performed on skeletal muscle lysates from subject 2 (p.Arg80His).
(B) Western blot analysis of inner mitochondrial membrane proteins performed on skeletal muscle samples from subject 5 (p.Arg235Gly).

from the genome was sufficient to sustain growth and suggesting that there was no dominant effect of the mutant proteins over the wild-type proteins at the transport level.

Additional investigations were undertaken to characterize the effect of the two mutations. Mitochondrial cytochrome content—an index of the structural integrity of the respiratory-chain complexes—was determined in the *aac2* (WB-12) strain carrying the *AAC2*, $aac2^{Arg96His}$, or $aac2^{Arg252Gly}$ alleles or the empty plasmid grown either at 28°C or at 37°C. The $aac2^{Arg96His}$ and $aac2^{Arg252Gly}$ mutations determined a significant reduction in absorption spectra at 37°C (Figure 5B). We also measured respiratory activity in the same transformants. At 37°C, oxygen consumption was decreased by 90% in both mutant strains compared to the wild-type *AAC2* strain (Figure 5C), in agreement with the lowered level of cytochromes and the growth phenotype on non-fermentable carbon sources.

To assess the ADP/ATP transport of the mutant carriers, transport assays were carried out for each mutation in both the WB-12 and W303-1B backgrounds. The p.Arg96His Aac2 mutant protein was expressed to a similar level as wild-type AAC2 in the WB-12 strain, but the ADP transport rate was only 60% of the wild-type (Figure 5D). This impairment of transport in yeast is not as great as for the human AAC1 p.Arg80His mutant expressed in the bacterial system described earlier (Figure 4B, 24%), suggesting a difference between the yeast and human proteins, which are only 52% identical. No transport activity was detected in the $aac2^{Arg252Gly}$ strain; however, this is likely due to the very low expression of the p.Arg252Gly Aac2 mutant protein as previously discussed (Figure 5D). Transport assays were also carried out with the wild-type and mutant carriers in the W303-1B background. Although the relative expression levels are not known, there seems to be no significant negative dominance effect on transport activity of the mutant proteins on the wild-type proteins (Figure 5D), corrected for protein levels, in agreement with earlier observations.[53]

AAC1 Alterations Cause a Dysregulation of Mitochondrial Protein Content

We next assessed another mechanism by which the genetic dominance of the de novo *SLC25A4* mutations could be explained, given the lack of a dominant-negative effect by in vitro experiments on the transport activity in yeast. A previous study modeling human mutation-associated adPEO in yeast suggested that the mutated Aac2 misfolds to place torsional stress on the mitochondrial inner membrane, leading to dysregulation of inner membrane proteins including loss of Oxa1p, a protein proposed to chaperone the insertion of proteins into the inner mitochondrial membrane, and of Tim22, which is involved in mitochondrial protein import.[54] We assessed steady-state levels of the human homologs OXA1L and TIMM22 in skeletal muscle samples from affected individuals but in contrast to the reports in yeast, OXA1L and TIMM22 were markedly increased in affected subjects relative to control subjects (Figure 6), suggesting that the lower levels of AAC1 in the affected individuals cannot be explained by loss of the protein import machinery.

Discussion

We describe seven subjects with de novo, heterozygous, single-nucleotide substitutions in *SLC25A4*; four subjects harbor a c.239G>A mutation predicted to substitute the arginine at position 80 to a histidine and three further subjects had a c.703C>G mutation predicted to substitute arginine 235 for a glycine residue. All affected individuals presented with severe congenital hypotonia and profound muscle weakness necessitating artificial ventilation, and several died in early infancy. Available muscle biopsies from affected individuals revealed histopathological evidence of mitochondrial myopathy and a severe combined respiratory chain defect as a consequence of marked depletion of mtDNA copy number (Table 1). The striking similarity in clinical phenotype across our cohort and the fact that the c.239G>A (p.Arg80His) or the c.703C>G (p.Arg235Gly) mutations have spontaneously arisen strongly suggest pathogenicity. Furthermore, both mutations affect highly conserved and functionally important amino acid residues, associated with loss of steady-state AAC1 levels in subjects' skeletal muscle and diminished ATP transport activity in vitro.

Mutations in *SLC25A4* are well-recognized causes of mitochondrial disease; dominantly inherited *SLC25A4* mutations have been documented in cases of adPEO, an

adulthood-onset mitochondrial disorder characterized by ptosis, restriction of eye movements, and the accumulation of clonally expanded mtDNA deletions in post-mitotic tissues on account of disordered mtDNA maintenance.[8–12] Additionally, recessively inherited *SLC25A4* mutations are known to cause a childhood or early adulthood-onset mitochondrial myopathy and cardiomyopathy phenotype with lactic acidosis and proximal muscle weakness[13–16] (summarized in Table S1). The case subjects we present here, with de novo dominant mutations, can be considered to show a third distinct clinical phenotype associated with mutations in *SLC25A4*, which is more severe in its clinical presentation than those previously described.

Despite the findings of *SLC25A4* mutations in adPEO with multiple mtDNA deletions,[8] the mechanism of how an ADP/ATP carrier causes mtDNA instability is unknown. Disorders of mtDNA maintenance are typically a consequence of mutations in replicative enzymes or those involved in nucleotide synthesis, leading to mtDNA depletion in the case of severe enzyme defects and multiple mtDNA deletions upon moderate defects.[55] The current finding of mtDNA depletion in early-onset cases with *SLC25A4* mutations links this gene to the causes of typical mtDNA maintenance disorders and strongly suggests that the transporter dysfunction causes insufficient adenine nucleotide availability for dATP synthesis and consequent imbalanced dNTP pools, leading to mtDNA depletion.

There has been considerable debate as to whether the dominant nature of *SLC25A4* mutations associated with adPEO is a consequence of dimerization of the carrier.[35,56] The de novo mutations we present also behave in a dominant manner, but both argue strongly against a dimer model as explanation for the dominance, because the affected residues point into the central cavity and thus cannot be directly involved in dimerization. This observation is consistent with studies carried out with the yeast mitochondrial ADP/ATP carrier, which have demonstrated that inactive carriers do not affect the function of active carriers.[53] Mitochondrial ADP/ATP carriers are structurally monomeric in the membrane[57] and in detergent,[46,48] and they do not have a plausible dimerization interface.[48] The monomer has all of the functional elements required for transport.[44,58,59] One verified exception for mitochondrial carriers is the mitochondrial aspartate/glutamate carrier, which dimerizes via its N-terminal regulatory domain.[60]

In an attempt to explain the differences in clinical presentation associated with the various reported *SLC25A4* mutations, we further analyzed the positions of affected amino acid residues within the structure of AAC1. Late-onset adPEO-associated mutations affect non-conserved parts of the carrier that do not have an obvious function, whereas the severe de novo mutations are both in highly conserved, functionally important parts of the carrier (Figure S3). Arg80 is contact point I in the proposed substrate binding site of the carrier (Figure 3C) and Arg235 forms one of the three salt bridge interactions required for formation of the matrix network (Figure 3D), predicting the de novo mutations to be more functionally detrimental to carrier function. Indeed, we have demonstrated that recombinant AAC1-Arg80His or AAC1-Arg235Gly mutants demonstrated severely impaired transport ability, with residual transport rates of only 24% and 3%, respectively (Figure 4B). The conservative p.Arg80His alteration impairs substrate binding to a large degree because histidine has a relatively short side chain and fewer potential conformers compared to arginine (Figure 3C). However, the large and flexible ADP molecule may adopt a binding pose that would be able to span the contact points of the substrate binding site, leading to a small amount of transport activity. The p.Arg235Gly substitution resulted in a lower residual transport activity of 3%, demonstrating the functional consequence of losing one of the three salt bridge interactions of the matrix network. The disruption and formation of the matrix network is a key part of the alternating access transport mechanism of AAC1.[51]

It is also interesting that residual transport activities broadly fall into three groups that segregate with the clinical phenotypes associated with different mutations. Mutations associated with adPEO manifested higher residual transport activities (24%–56%) than the de novo mutations, whereas the mutations associated with recessive disease were completely null (<1% transport activity) (Figure 4B). In the cases of the dominant mutations, the severity of transport activity impairment correlates with the severity of the clinical phenotypes. The majority of adPEO-associated mutations showed approximately 50% residual transport activity (Figure 4B). Assuming equal expression of wild-type and mutant transporters, the total flux across the inner membrane would be ≈75% compared to healthy individuals. It is likely that there is spare capacity in transport in order to deal with changes in bioenergetic demand (e.g., rest versus exercise). In the case of the de novo mutations, the mutant transporters have lower residual transport activities (3%–24%) (Figure 4B) and lower expression levels (Figures 2A and 2C) resulting in an energy crisis, which in turn likely affects many other key mitochondrial functions leading to the severe, early-onset, and often fatal clinical presentations.

Reports of recessive *SLC25A4* mutations do not fit with a correlation of AAC1 transport ability and clinical severity, since the mutations (including nonsense,[15,16] splicing,[14] and missense[13,16]) are completely null but have a much milder clinical phenotype than the case subjects presented here (Tables 1 and S1). We confirmed that each of the recessive missense mutations produce inactive transporters (Figure 4B). In agreement, the affected residues are also pointing inward and thus possibly interfere with key functions of the carrier (Figure S3). Knockout mouse studies have also shown that loss of AAC1 produces a similar myopathy and cardiomyopathy phenotype to individuals with recessive *SLC25A4* mutations.[61,62] We therefore

postulate that the complete lack of functional AAC1 triggers a compensatory mechanism to upregulate expression of other isoforms of ADP/ATP carrier in cases of recessive disease. There are four different AAC isoforms and at least another three isoforms of the ATP-Mg/Pi carrier, which can also transport ADP and ATP.[63] All current information on the relative expression levels in different tissues is based on RNA studies.[17] At present it is not possible to study the relative expression of wild-type and mutant AAC1 at the protein level nor are we able to distinguish between each AAC isoform since the proteins are so similar (>88% identity between AAC1, AAC2, and AAC3) that the available antibodies recognize all isoforms.

The Arg252Gly mutant was expressed to very low levels in yeast mitochondria (Figures 5C and 5D). This observation may indicate that residues of the matrix salt bridge network might have additional functions in the biogenesis of the carrier, which could also explain the apparent decrease in AAC1 protein levels seen in skeletal muscle from subject 5 (Figure 2B). However, AAC1 protein levels were also depleted in subject 1 (Figure 2A), but the expression of the equivalent Arg96His mutant in yeast was comparable to wild-type Aac2 (Figures 5C and 5D). In all tested subjects, residual AAC steady-state levels were approximately 10%–30% (Figure 2), which is surprising given the heterozygous nature of these mutations. We investigated whether loss of AAC1 could be explained by misfolding of the protein, which may exert torsional stress on the mitochondrial inner membrane and disrupt the function of other mitochondrial proteins, which has been shown with mutations in yeast that model some of the human mutations associated with adPEO.[54] In yeast strains expressing mutated Aac2, steady-state levels of other inner membrane proteins such as Oxa1p and Tim22 were decreased.[54] Western blot analysis of subject muscle tissue actually showed a marked increase in OXA1L and TIMM22 protein levels (Figures 6A and 6B), suggesting a different mechanism. However, the increase in OXA1L relative to other inner mitochondrial membrane proteins and the disorganized cristae observed by electron microscopy in subject 3 (Figure S1) does suggest that the AAC1 mutations, and the consequent effect on expression levels, has a profound effect on inner membrane content and structure. AAC1 is the most abundant protein of the inner mitochondrial membrane in skeletal muscle and disruption of its expression may explain the observed morphological changes.

To summarize the clinical heterogeneity of *SLC25A4* mutations, we hypothesize that adPEO mutations are less severe due to the mutations affecting peripheral amino acids of AAC1 not crucial for transport; recessively inherited mutations are null for AAC1 transport ability and another transporter is likely upregulated to compensate; de novo dominant mutations are the most clinically severe as they affect amino acids in crucial functional domains of AAC1, resulting in severely impaired transport ability and a decrease in total AAC expression, thus ruling out compensation from other isoforms possibly due to effects on insertion and stability of the protein.

The recurrent nature of the de novo c.239G>A (p.Arg80His) mutation is likely due to it being located within a CpG dinucleotide, which is a known mutation hotspot.[64] Mutation rates at CpG sites are estimated to be 18.2 times higher than at non-CpG sites due to spontaneous deamination of 5-methylcytosine nucleotides to thymine.[65] Most reports of recurrent de novo mutations associated with disease occur in CpG sites causing substitution of arginine residues.[66–70] The c.703C>G (p.Arg235Gly) mutation is not located at a CpG site, which may explain the less frequent recurrence.

Importantly, the recurrent nature of these mutations suggests that pathogenic de novo mutations may be far more common causes of early-onset mitochondrial disease than anticipated. Confirming the pathogenicity of these de novo mutations has immense implications for the application of WES, and the subsequent filtering of sequence data, to the investigation of mitochondrial disease; all severe, early-onset Mendelian cases reported to date show a recessive pattern of inheritance and as such a recessive model of disease is prioritized in bioinformatic pipelines. WES of family trios (parents and proband) allows de novo mutations to be easily identified as candidates for further functional studies. Our study underscores the importance of sequencing trios: subjects 1, 6, and 7 were initially sequenced as singleton case subjects, remaining unresolved until the subsequent identification of the mutations as de novo and present in other affected individuals with identical clinical phenotypes.

Accession Numbers

The ClinVar accession number for c.703C>G (p.Arg235Gly) is SCV000297810 and for c.239G>A (p.Arg80His) is SCV000297809.

Supplemental Data

Supplemental Data include three figures and one table and can be found with this article online at http://dx.doi.org/10.1016/j.ajhg.2016.08.014.

Acknowledgments

This work was supported by a Wellcome Trust Strategic Award (096919/Z/11/Z), the MRC Centre for Neuromuscular Diseases (G0601943), the Lily Foundation, the UK NHS Highly Specialised "Rare Mitochondrial Disorders of Adults and Children" Service in Newcastle upon Tyne, an NIHR/CSO Healthcare Science Research Fellowship from the National Institute for Health Research (NIHR-HCS-D12-03-04 to C.L.A.), the Department of Health via the NIHR comprehensive Biomedical Research Centre award to Guy's and St. Thomas' NHS Foundation Trust in partnership with the King's College London, Medical Research Council UK (MC_U105663139) (E.R.S.K., M.S.K., H.M.), Fondazione Telethon (Italy) (grant GGP15041) (C. Dallabona, T.L., I.F.), Estonian Science Foundation (grants 8175 and PUT355) (K.R., K.Õ.), Jane and Aatos Erkko Foundation, Sigrid Jusélius Foundation, Academy

of Finland and Helsinki University Hospital Funds (P.I., A. Suomalainen), and National Institute of Neurological Disorders and Stroke of the National Institutes of Health (R01NS083726) (P.E.B.). We would also like to thank the Genome Technology Center at the RadboudUMC and BGI Copenhagen (R.J.R.) and Institute of Molecular Medicine Finland (A. Suomalainen) for technical support of the exome sequencing, together with colleagues at the cell culture, muscle, and DNA laboratories of the Translational Metabolic Laboratory, RadboudUMC Nijmegen for excellent technical assistance (R.J.R.).

Received: June 24, 2016
Accepted: August 18, 2016
Published: September 29, 2016

Web Resources

ClinVar, https://www.ncbi.nlm.nih.gov/clinvar/
ExAC Browser, http://exac.broadinstitute.org/
GenBank, http://www.ncbi.nlm.nih.gov/genbank/
OMIM, http://www.omim.org/
PolyPhen-2, http://genetics.bwh.harvard.edu/pph2/
RRID, https://scicrunch.org/resources
SIFT, http://sift.bii.a-star.edu.sg/
SISu Project, http://www.sisuproject.fi/

References

1. Anderson, S., Bankier, A.T., Barrell, B.G., de Bruijn, M.H., Coulson, A.R., Drouin, J., Eperon, I.C., Nierlich, D.P., Roe, B.A., Sanger, F., et al. (1981). Sequence and organization of the human mitochondrial genome. Nature *290*, 457–465.
2. Lightowlers, R.N., Taylor, R.W., and Turnbull, D.M. (2015). Mutations causing mitochondrial disease: What is new and what challenges remain? Science *349*, 1494–1499.
3. Taylor, R.W., Pyle, A., Griffin, H., Blakely, E.L., Duff, J., He, L., Smertenko, T., Alston, C.L., Neeve, V.C., Best, A., et al. (2014). Use of whole-exome sequencing to determine the genetic basis of multiple mitochondrial respiratory chain complex deficiencies. JAMA *312*, 68–77.
4. Kunji, E.R.S. (2004). The role and structure of mitochondrial carriers. FEBS Lett. *564*, 239–244.
5. Saraste, M., and Walker, J.E. (1982). Internal sequence repeats and the path of polypeptide in mitochondrial ADP/ATP translocase. FEBS Lett. *144*, 250–254.
6. Palmieri, F. (2014). Mitochondrial transporters of the SLC25 family and associated diseases: a review. J. Inherit. Metab. Dis. *37*, 565–575.
7. Klingenberg, M. (2008). The ADP and ATP transport in mitochondria and its carrier. Biochim. Biophys. Acta *1778*, 1978–2021.
8. Kaukonen, J., Juselius, J.K., Tiranti, V., Kyttälä, A., Zeviani, M., Comi, G.P., Keränen, S., Peltonen, L., and Suomalainen, A. (2000). Role of adenine nucleotide translocator 1 in mtDNA maintenance. Science *289*, 782–785.
9. Napoli, L., Bordoni, A., Zeviani, M., Hadjigeorgiou, G.M., Sciacco, M., Tiranti, V., Terentiou, A., Moggio, M., Papadimitriou, A., Scarlato, G., and Comi, G.P. (2001). A novel missense adenine nucleotide translocator-1 gene mutation in a Greek adPEO family. Neurology *57*, 2295–2298.
10. Komaki, H., Fukazawa, T., Houzen, H., Yoshida, K., Nonaka, I., and Goto, Y. (2002). A novel D104G mutation in the adenine nucleotide translocator 1 gene in autosomal dominant progressive external ophthalmoplegia patients with mitochondrial DNA with multiple deletions. Ann. Neurol. *51*, 645–648.
11. Siciliano, G., Tessa, A., Petrini, S., Mancuso, M., Bruno, C., Grieco, G.S., Malandrini, A., DeFlorio, L., Martini, B., Federico, A., et al. (2003). Autosomal dominant external ophthalmoplegia and bipolar affective disorder associated with a mutation in the ANT1 gene. Neuromuscul. Disord. *13*, 162–165.
12. Deschauer, M., Hudson, G., Müller, T., Taylor, R.W., Chinnery, P.F., and Zierz, S. (2005). A novel ANT1 gene mutation with probable germline mosaicism in autosomal dominant progressive external ophthalmoplegia. Neuromuscul. Disord. *15*, 311–315.
13. Palmieri, L., Alberio, S., Pisano, I., Lodi, T., Meznaric-Petrusa, M., Zidar, J., Santoro, A., Scarcia, P., Fontanesi, F., Lamantea, E., et al. (2005). Complete loss-of-function of the heart/muscle-specific adenine nucleotide translocator is associated with mitochondrial myopathy and cardiomyopathy. Hum. Mol. Genet. *14*, 3079–3088.
14. Echaniz-Laguna, A., Chassagne, M., Ceresuela, J., Rouvet, I., Padet, S., Acquaviva, C., Nataf, S., Vinzio, S., Bozon, D., and Mousson de Camaret, B. (2012). Complete loss of expression of the ANT1 gene causing cardiomyopathy and myopathy. J. Med. Genet. *49*, 146–150.
15. Strauss, K.A., DuBiner, L., Simon, M., Zaragoza, M., Sengupta, P.P., Li, P., Narula, N., Dreike, S., Platt, J., Procaccio, V., et al. (2013). Severity of cardiomyopathy associated with adenine nucleotide translocator-1 deficiency correlates with mtDNA haplogroup. Proc. Natl. Acad. Sci. USA *110*, 3453–3458.
16. Körver-Keularts, I.M.L.W., de Visser, M., Bakker, H.D., Wanders, R.J.A., Vansenne, F., Scholte, H.R., Dorland, L., Nicolaes, G.A.F., Spaapen, L.M.J., Smeets, H.J.M., et al. (2015). Two novel mutations in the *SLC25A4* gene in a patient with mitochondrial myopathy. JIMD Rep. *22*, 39–45.
17. Dolce, V., Scarcia, P., Iacopetta, D., and Palmieri, F. (2005). A fourth ADP/ATP carrier isoform in man: identification, bacterial expression, functional characterization and tissue distribution. FEBS Lett. *579*, 633–637.
18. Li, K., Warner, C.K., Hodge, J.A., Minoshima, S., Kudoh, J., Fukuyama, R., Maekawa, M., Shimizu, Y., Shimizu, N., and Wallace, D.C. (1989). A human muscle adenine nucleotide translocator gene has four exons, is located on chromosome 4, and is differentially expressed. J. Biol. Chem. *264*, 13998–14004.
19. Clémençon, B., Babot, M., and Trézéguet, V. (2013). The mitochondrial ADP/ATP carrier (SLC25 family): pathological implications of its dysfunction. Mol. Aspects Med. *34*, 485–493.
20. Kirby, D.M., Thorburn, D.R., Turnbull, D.M., and Taylor, R.W. (2007). Biochemical assays of respiratory chain complex activity. Methods Cell Biol. *80*, 93–119.
21. Rodenburg, R.J.T. (2011). Biochemical diagnosis of mitochondrial disorders. J. Inherit. Metab. Dis. *34*, 283–292.
22. Majander, A., Rapola, J., Sariola, H., Suomalainen, A., Pohjavuori, M., and Pihko, H. (1995). Diagnosis of fatal infantile defects of the mitochondrial respiratory chain: age dependence and postmortem analysis of enzyme activities. J. Neurol. Sci. *134*, 95–102.
23. Simpson, M.A., Irving, M.D., Asilmaz, E., Gray, M.J., Dafou, D., Elmslie, F.V., Mansour, S., Holder, S.E., Brain, C.E., Burton, B.K., et al. (2011). Mutations in *NOTCH2* cause Hajdu-Cheney syndrome, a disorder of severe and progressive bone loss. Nat. Genet. *43*, 303–305.

24. Neveling, K., Feenstra, I., Gilissen, C., Hoefsloot, L.H., Kamsteeg, E.-J., Mensenkamp, A.R., Rodenburg, R.J.T., Yntema, H.G., Spruijt, L., Vermeer, S., et al. (2013). A post-hoc comparison of the utility of sanger sequencing and exome sequencing for the diagnosis of heterogeneous diseases. Hum. Mutat. *34*, 1721–1726.
25. Wortmann, S.B., Koolen, D.A., Smeitink, J.A., van den Heuvel, L., and Rodenburg, R.J. (2015). Whole exome sequencing of suspected mitochondrial patients in clinical practice. J. Inherit. Metab. Dis. *38*, 437–443.
26. Mandel, H., Saita, S., Edvardson, S., Jalas, C., Shaag, A., Goldsher, D., Vlodavsky, E., Langer, T., and Elpeleg, O. (2016). Deficiency of HTRA2/Omi is associated with infantile neurodegeneration and 3-methylglutaconic aciduria. J. Med. Genet. Published online May 12, 2016. http://dx.doi.org/10.1136/jmedgenet-2016-103922.
27. Sulonen, A.-M., Ellonen, P., Almusa, H., Lepistö, M., Eldfors, S., Hannula, S., Miettinen, T., Tyynismaa, H., Salo, P., Heckman, C., et al. (2011). Comparison of solution-based exome capture methods for next generation sequencing. Genome Biol. *12*, R94.
28. Carroll, C.J., Isohanni, P., Pöyhönen, R., Euro, L., Richter, U., Brilhante, V., Götz, A., Lahtinen, T., Paetau, A., Pihko, H., et al. (2013). Whole-exome sequencing identifies a mutation in the mitochondrial ribosome protein MRPL44 to underlie mitochondrial infantile cardiomyopathy. J. Med. Genet. *50*, 151–159.
29. Ho, S.N., Hunt, H.D., Horton, R.M., Pullen, J.K., and Pease, L.R. (1989). Site-directed mutagenesis by overlap extension using the polymerase chain reaction. Gene *77*, 51–59.
30. de Ruyter, P.G., Kuipers, O.P., and de Vos, W.M. (1996). Controlled gene expression systems for *Lactococcus lactis* with the food-grade inducer nisin. Appl. Environ. Microbiol. *62*, 3662–3667.
31. Kunji, E.R.S., Chan, K.W., Slotboom, D.J., Floyd, S., O'Connor, R., and Monné, M. (2005). Eukaryotic membrane protein overproduction in *Lactococcus lactis*. Curr. Opin. Biotechnol. *16*, 546–551.
32. Thomas, B.J., and Rothstein, R. (1989). Elevated recombination rates in transcriptionally active DNA. Cell *56*, 619–630.
33. Hashimoto, M., Shinohara, Y., Majima, E., Hatanaka, T., Yamazaki, N., and Terada, H. (1999). Expression of the bovine heart mitochondrial ADP/ATP carrier in yeast mitochondria: significantly enhanced expression by replacement of the N-terminal region of the bovine carrier by the corresponding regions of the yeast carriers. Biochim. Biophys. Acta *1409*, 113–124.
34. Baruffini, E., Ferrero, I., and Foury, F. (2010). In vivo analysis of mtDNA replication defects in yeast. Methods *51*, 426–436.
35. Fontanesi, F., Palmieri, L., Scarcia, P., Lodi, T., Donnini, C., Limongelli, A., Tiranti, V., Zeviani, M., Ferrero, I., and Viola, A.M. (2004). Mutations in *AAC2*, equivalent to human adPEO-associated ANT1 mutations, lead to defective oxidative phosphorylation in Saccharomyces cerevisiae and affect mitochondrial DNA stability. Hum. Mol. Genet. *13*, 923–934.
36. Bonneaud, N., Ozier-Kalogeropoulos, O., Li, G.Y., Labouesse, M., Minvielle-Sebastia, L., and Lacroute, F. (1991). A family of low and high copy replicative, integrative and single-stranded *S. cerevisiae*/*E. coli* shuttle vectors. Yeast *7*, 609–615.
37. Gietz, R.D., and Schiestl, R.H. (2007). High-efficiency yeast transformation using the LiAc/SS carrier DNA/PEG method. Nat. Protoc. *2*, 31–34.
38. Goffrini, P., Ercolino, T., Panizza, E., Giachè, V., Cavone, L., Chiarugi, A., Dima, V., Ferrero, I., and Mannelli, M. (2009). Functional study in a yeast model of a novel succinate dehydrogenase subunit B gene germline missense mutation (C191Y) diagnosed in a patient affected by a glomus tumor. Hum. Mol. Genet. *18*, 1860–1868.
39. Barrientos, A. (2002). In vivo and in organello assessment of OXPHOS activities. Methods *26*, 307–316.
40. Adzhubei, I., Jordan, D.M., and Sunyaev, S.R. (2013). Predicting functional effect of human missense mutations using PolyPhen-2. Curr. Protoc. Hum. Genet. *Chapter 7*, 20.
41. Kumar, P., Henikoff, S., and Ng, P.C. (2009). Predicting the effects of coding non-synonymous variants on protein function using the SIFT algorithm. Nat. Protoc. *4*, 1073–1081.
42. Kircher, M., Witten, D.M., Jain, P., O'Roak, B.J., Cooper, G.M., and Shendure, J. (2014). A general framework for estimating the relative pathogenicity of human genetic variants. Nat. Genet. *46*, 310–315.
43. Tavtigian, S.V., Deffenbaugh, A.M., Yin, L., Judkins, T., Scholl, T., Samollow, P.B., de Silva, D., Zharkikh, A., and Thomas, A. (2006). Comprehensive statistical study of 452 BRCA1 missense substitutions with classification of eight recurrent substitutions as neutral. J. Med. Genet. *43*, 295–305.
44. Robinson, A.J., Overy, C., and Kunji, E.R.S. (2008). The mechanism of transport by mitochondrial carriers based on analysis of symmetry. Proc. Natl. Acad. Sci. USA *105*, 17766–17771.
45. Nelson, D.R., Felix, C.M., and Swanson, J.M. (1998). Highly conserved charge-pair networks in the mitochondrial carrier family. J. Mol. Biol. *277*, 285–308.
46. Pebay-Peyroula, E., Dahout-Gonzalez, C., Kahn, R., Trézéguet, V., Lauquin, G.J.-M., and Brandolin, G. (2003). Structure of mitochondrial ADP/ATP carrier in complex with carboxyatractyloside. Nature *426*, 39–44.
47. King, M.S., Kerr, M., Crichton, P.G., Springett, R., and Kunji, E.R.S. (2016). Formation of a cytoplasmic salt bridge network in the matrix state is a fundamental step in the transport mechanism of the mitochondrial ADP/ATP carrier. Biochim. Biophys. Acta *1857*, 14–22.
48. Ruprecht, J.J., Hellawell, A.M., Harding, M., Crichton, P.G., McCoy, A.J., and Kunji, E.R.S. (2014). Structures of yeast mitochondrial ADP/ATP carriers support a domain-based alternating-access transport mechanism. Proc. Natl. Acad. Sci. USA *111*, E426–E434.
49. Kunji, E.R.S., and Robinson, A.J. (2006). The conserved substrate binding site of mitochondrial carriers. Biochim. Biophys. Acta *1757*, 1237–1248.
50. Robinson, A.J., and Kunji, E.R.S. (2006). Mitochondrial carriers in the cytoplasmic state have a common substrate binding site. Proc. Natl. Acad. Sci. USA *103*, 2617–2622.
51. Kunji, E.R.S., Aleksandrova, A., King, M.S., Majd, H., Ashton, V.L., Cerson, E., Springett, R., Kibalchenko, M., Tavoulari, S., Crichton, P.G., and Ruprecht, J.J. (2016). The transport mechanism of the mitochondrial ADP/ATP carrier. Biochim. Biophys. Acta *1863*, 2379–2393. http://dx.doi.org/10.1016/j.bbamcr.2016.03.015.
52. Mifsud, J., Ravaud, S., Krammer, E.-M., Chipot, C., Kunji, E.R.S., Pebay-Peyroula, E., and Dehez, F. (2013). The substrate specificity of the human ADP/ATP carrier AAC1. Mol. Membr. Biol. *30*, 160–168.
53. Bamber, L., Harding, M., Monné, M., Slotboom, D.J., and Kunji, E.R. (2007). The yeast mitochondrial ADP/ATP carrier

functions as a monomer in mitochondrial membranes. Proc. Natl. Acad. Sci. USA *104*, 10830–10834.

54. Liu, Y., Wang, X., and Chen, X.J. (2015). Misfolding of mutant adenine nucleotide translocase in yeast supports a novel mechanism of Ant1-induced muscle diseases. Mol. Biol. Cell *26*, 1985–1994. http://dx.doi.org/10.1091/mbc.E15-01-0030.
55. Suomalainen, A., and Isohanni, P. (2010). Mitochondrial DNA depletion syndromes–many genes, common mechanisms. Neuromuscul. Disord. *20*, 429–437.
56. Liu, Y., and Chen, X.J. (2013). Adenine nucleotide translocase, mitochondrial stress, and degenerative cell death. Oxid. Med. Cell. Longev. *2013*, 146860. http://dx.doi.org/10.1155/2013/146860.
57. Kunji, E.R.S., and Harding, M. (2003). Projection structure of the atractyloside-inhibited mitochondrial ADP/ATP carrier of *Saccharomyces cerevisiae*. J. Biol. Chem. *278*, 36985–36988.
58. Kunji, E.R.S., and Crichton, P.G. (2010). Mitochondrial carriers function as monomers. Biochim. Biophys. Acta *1797*, 817–831.
59. Crichton, P.G., Harding, M., Ruprecht, J.J., Lee, Y., and Kunji, E.R.S. (2013). Lipid, detergent, and Coomassie Blue G-250 affect the migration of small membrane proteins in blue native gels: mitochondrial carriers migrate as monomers not dimers. J. Biol. Chem. *288*, 22163–22173.
60. Thangaratnarajah, C., Ruprecht, J.J., and Kunji, E.R.S. (2014). Calcium-induced conformational changes of the regulatory domain of human mitochondrial aspartate/glutamate carriers. Nat. Commun. *5*, 5491.
61. Graham, B.H., Waymire, K.G., Cottrell, B., Trounce, I.A., MacGregor, G.R., and Wallace, D.C. (1997). A mouse model for mitochondrial myopathy and cardiomyopathy resulting from a deficiency in the heart/muscle isoform of the adenine nucleotide translocator. Nat. Genet. *16*, 226–234.
62. Esposito, L.A., Melov, S., Panov, A., Cottrell, B.A., and Wallace, D.C. (1999). Mitochondrial disease in mouse results in increased oxidative stress. Proc. Natl. Acad. Sci. USA *96*, 4820–4825.
63. Fiermonte, G., De Leonardis, F., Todisco, S., Palmieri, L., Lasorsa, F.M., and Palmieri, F. (2004). Identification of the mitochondrial ATP-Mg/Pi transporter. Bacterial expression, reconstitution, functional characterization, and tissue distribution. J. Biol. Chem. *279*, 30722–30730.
64. Coulondre, C., Miller, J.H., Farabaugh, P.J., and Gilbert, W. (1978). Molecular basis of base substitution hotspots in *Escherichia coli*. Nature *274*, 775–780.
65. Kong, A., Frigge, M.L., Masson, G., Besenbacher, S., Sulem, P., Magnusson, G., Gudjonsson, S.A., Sigurdsson, A., Jonasdottir, A., Jonasdottir, A., et al. (2012). Rate of de novo mutations and the importance of father's age to disease risk. Nature *488*, 471–475.
66. Wagnon, J.L., and Meisler, M.H. (2015). Recurrent and non-recurrent mutations of *SCN8A* in epileptic encephalopathy. Front. Neurol. *6*, 104.
67. Muona, M., Berkovic, S.F., Dibbens, L.M., Oliver, K.L., Maljevic, S., Bayly, M.A., Joensuu, T., Canafoglia, L., Franceschetti, S., Michelucci, R., et al. (2015). A recurrent de novo mutation in *KCNC1* causes progressive myoclonus epilepsy. Nat. Genet. *47*, 39–46.
68. Kearney, J.A., Wiste, A.K., Stephani, U., Trudeau, M.M., Siegel, A., RamachandranNair, R., Elterman, R.D., Muhle, H., Reinsdorf, J., Shields, W.D., et al. (2006). Recurrent de novo mutations of *SCN1A* in severe myoclonic epilepsy of infancy. Pediatr. Neurol. *34*, 116–120.
69. Schuurs-Hoeijmakers, J.H.M., Oh, E.C., Vissers, L.E.L.M., Swinkels, M.E.M., Gilissen, C., Willemsen, M.A., Holvoet, M., Steehouwer, M., Veltman, J.A., de Vries, B.B.A., et al. (2012). Recurrent de novo mutations in *PACS1* cause defective cranial-neural-crest migration and define a recognizable intellectual-disability syndrome. Am. J. Hum. Genet. *91*, 1122–1127.
70. Fischer-Zirnsak, B., Escande-Beillard, N., Ganesh, J., Tan, Y.X., Al Bughaili, M., Lin, A.E., Sahai, I., Bahena, P., Reichert, S.L., Loh, A., et al. (2015). Recurrent de novo mutations affecting residue Arg138 of pyrroline-5-carboxylate synthase cause a progeroid form of autosomal-dominant cutis laxa. Am. J. Hum. Genet. *97*, 483–492.

The American Journal of Human Genetics, Volume *99*

Supplemental Data

Recurrent De Novo Dominant Mutations in *SLC25A4* Cause Severe Early-Onset Mitochondrial Disease and Loss of Mitochondrial DNA Copy Number

Kyle Thompson, Homa Majd, Christina Dallabona, Karit Reinson, Martin S. King, Charlotte L. Alston, Langping He, Tiziana Lodi, Simon A. Jones, Aviva Fattal-Valevski, Nitay D. Fraenkel, Ann Saada, Alon Haham, Pirjo Isohanni, Roshni Vara, Inês A. Barbosa, Michael A. Simpson, Charu Deshpande, Sanna Puusepp, Penelope E. Bonnen, Richard J. Rodenburg, Anu Suomalainen, Katrin Õunap, Orly Elpeleg, Ileana Ferrero, Robert McFarland, Edmund R.S. Kunji, and Robert W. Taylor

Supplemental Information

Supplemental Figures

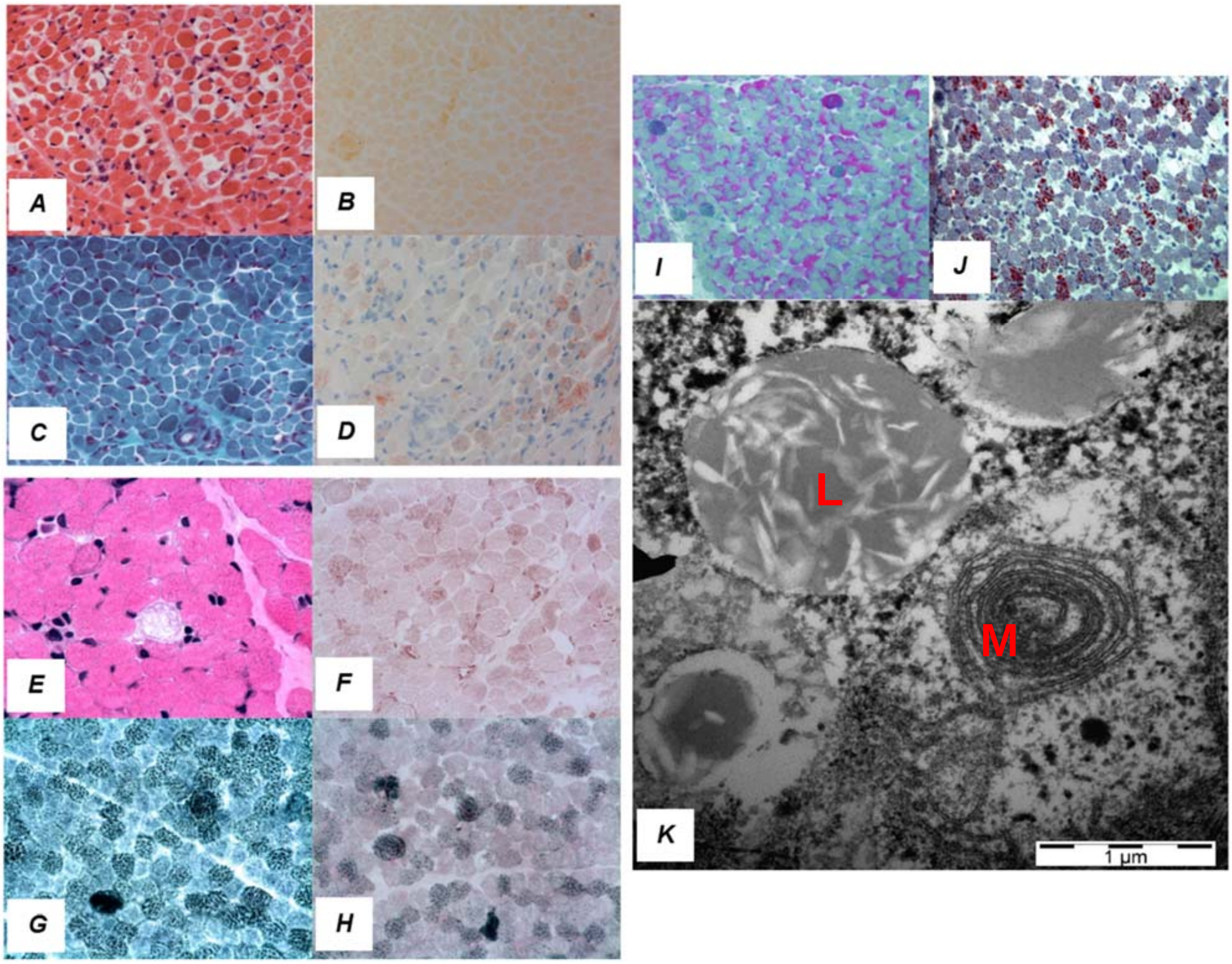

Figure S1: Muscle histopathology and electron microscopy in subjects with de novo heterozygous c.239G>A, p.(Arg80His) *SLC25A4* mutation
Histopathological analysis of a serially-sectioned skeletal muscle biopsy from Subject 1 showing (***A***) H&E staining, (***B***) cytochrome *c* oxidase (COX) reaction, (***C***) modified Gomori Trichrome staining and (***D***) Oil Red O staining highlighting evidence of weak COX reactivity across the section and accumulation of neutral lipid. Similar findings are evident in subject 2 based on (***E***) H&E staining, (***F***) cytochrome *c* oxidase (COX) reaction, (***G***) succinate dehydrogenase reaction showing fibres with enhanced mitochondrial numbers and (***H***) Sudan black staining to identify lipid deposition. Whilst oxidative enzyme reactions were not undertaken in subject 3, (***I***) modified Gomori trichrome staining and (***J***) Oil Red O staining show abnormal accumulations respectively. (***K***) An electron micrograph shows abnormal mitochondria with circularly arranged cristae (M) and lipid droplets (L).

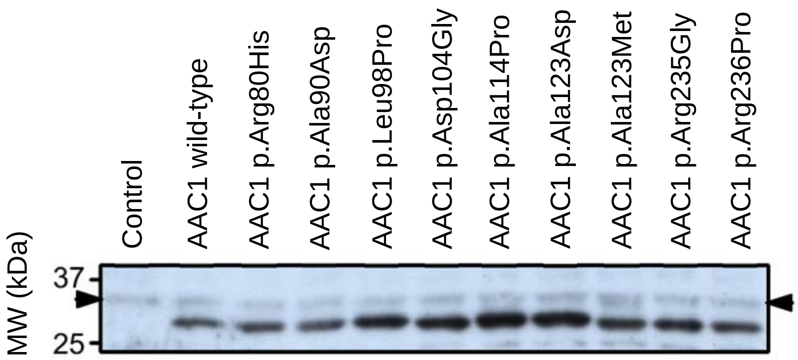

Figure S2: Recombinant AAC1 mutant proteins expressed in *L. lactis*
Western blot showing expression of the various human AAC1 mutants associated with disease. The arrow head shows a non-specific band, which serves as a loading control.

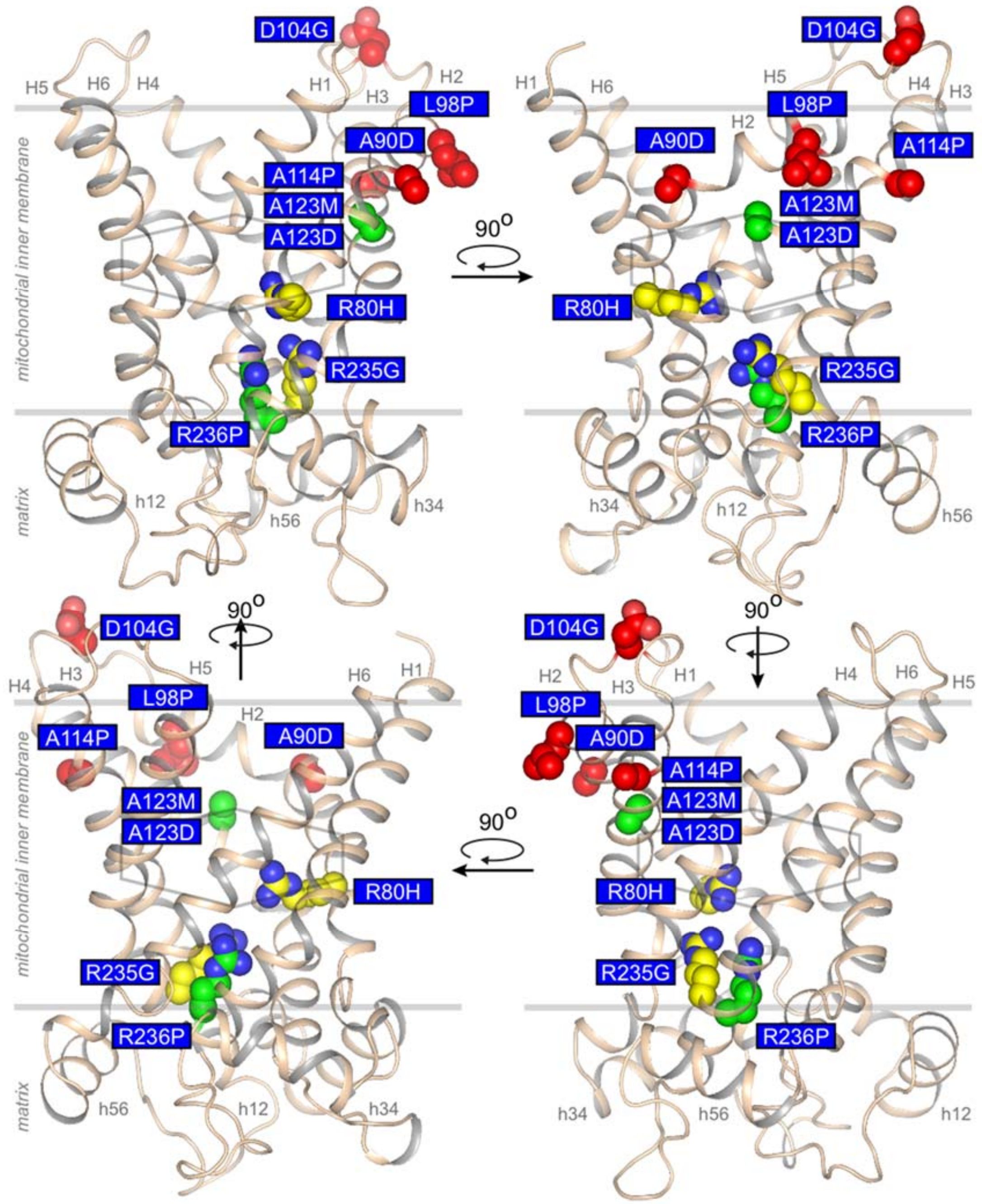

Figure S3: Position of disease-associated AAC1 mutations
Lateral view of the human ADP/ATP carrier from the membrane, showing the location of residues found to be mutated in disease. The *de novo* mutations are shown in yellow, dominant adPEO mutations in red and the recessive mutations in green.

Supplemental Table

Table S1: Previously reported *SLC25A4* mutations and associated clinical phenotype			
**Inheritance**	**Mutation**	**Clinical Phenotype**	**Reference**
Dominant	c.[342 G>C] + [=]<br>p.[(Ala114Pro)] + [=]	adPEO	Kaukonen *et al.* 2000
Dominant	c.[867 G>A] + [=]<br>p.[(Val289Met)] + [=]	adPEO	Kaukonen *et al.* 2000
Dominant	c.[293T>C] + [=]<br>p.[(Leu98Pro)] + [=]	adPEO	Napoli *et al.* 2001
Dominant	c.[311A>G] + [=]<br>p.[(Asp104Gly)] + [=]	adPEO	Komaki *et al.* 2002
Dominant	c.[293T>C] + [=]<br>p.[(Leu98Pro)] + [=]	adPEO,	Siciliano *et al.* 2003
Dominant	c.[269C>A] + [=]<br>p.[(Ala90Asp)] + [=]	adPEO	Deschauer *et al.* 2005
Recessive	c.[368C>A] + c.[368C>A]<br>p.[(Ala123Asp)] + p.[(Ala123Asp)]	Mitochondrial myopathy and cardiomyopathy	Palmieri *et al.* 2005
Recessive	c.[111+1G>A] + c.[111+1G>A]<br>(abolishes GT splice donor site of intron 1)	Mitochondrial myopathy and cardiomyopathy	Echaniz-Laguna *et al.* 2012
Recessive	c.[523delC] + c.[523delC]<br>p.[(Gln175ArgfsX38)] + p.[(Gln175ArgfsX38)]	Mitochondrial myopathy and cardiomyopathy	Strauss *et al.* 2013
Recessive	c.[707G>C] + c.[116-137del]<br>p.[(Arg236Pro)] + p.[(Gln39Leufs*14)]	Mitochondrial myopathy and cardiomyopathy	Korver-Keularts *et al.* 2015

Supplemental References:

Kaukonen, J. et al. Role of adenine nucleotide translocator 1 in mtDNA maintenance. *Science* 2000:**289**, 782–785.

Napoli, L. et al. A novel missense adenine nucleotide translocator-1 gene mutation in a Greek adPEO family. *Neurology* 2001:**57**, 2295–2298.

Komaki, H. et al. A novel D104G mutation in the adenine nucleotide translocator 1 gene in autosomal dominant progressive external ophthalmoplegia patients with mitochondrial DNA with multiple deletions. *Ann Neurol*. 2002:**51**, 645–648.

Siciliano, G. et al. Autosomal dominant external ophthalmoplegia and bipolar affective disorder associated with a mutation in the ANT1 gene. *Neuromuscul. Disord*. 2003:**13**, 162–165.

Deschauer, M. et al. A novel ANT1 gene mutation with probable germline mosaicism in autosomal dominant progressive external ophthalmoplegia. *Neuromuscul. Disord.* 2005:**15**, 311–315.

Palmieri, L. et al. Complete loss-of-function of the heart/muscle-specific adenine nucleotide translocator is associated with mitochondrial myopathy and cardiomyopathy. *Hum Mol Genet* 2005:**14**, 3079–3088.

Echaniz-Laguna, A. et al. Complete loss of expression of the ANT1 gene causing cardiomyopathy and myopathy. *Journal of Medical Genetics* 2012:**49**, 146–150.

Strauss, K.A. et al. Severity of cardiomyopathy associated with adenine nucleotide translocator-1 deficiency correlates with mtDNA haplogroup. *PNAS* 2013:**110**, 3453–3458.

Körver-Keularts, I.M.L.W. et al. Two Novel Mutations in the SLC25A4 Gene in a Patient with Mitochondrial Myopathy. *JIMD Rep* 2015:**22**:39-
